# Physics-Guided Decision-Support Framework for Melt Pool Prediction and Process Stability in Laser Powder Bed Fusion of Nitinol

**DOI:** 10.3390/ma19173696

**Published:** 2026-08-30

**Authors:** Sampreet Rangaswamy, Merve Nur Doğu, Camille Rubio, Hengfeng Gu, Abdul Khader Khan, Chong Teng, Inam Ul Ahad, Dermot Brabazon

**Affiliations:** 1 I-Form, Advanced Manufacturing Research Centre, Dublin City University, Glasnevin, D09 V209 Dublin, Ireland; mervenur.dogu@dcu.ie (M.N.D.); inamul.ahad@dcu.ie (I.U.A.); dermot.brabazon@dcu.ie (D.B.); 2 DCU Institute for Advanced Processing Technology Research Centre, School of Mechanical and Manufacturing Engineering, Dublin City University, Glasnevin, D09 V209 Dublin, Ireland; 3 Polytech Paris-Saclay, Université Paris-Saclay, 91405 Orsay CEDEX, France; 4 Ansys Inc., 6975 Union Park Avenue, Suite 663, Cottonwood Heights, UT 84047, USA; hengfeng.gu@synopsys.com (H.G.); chong.teng@synopsys.com (C.T.)

**Keywords:** NiTi, powder bed fusion-laser beam, thermal finite element modeling, CALPHAD, machine learning, digital twin

## Abstract

Powder bed fusion–laser beam (PBF-LB) of nickel–titanium (NiTi) has attracted increasing interest in aerospace, biomedical, and energy applications owing to its shape memory and superelastic properties, combined with the capability to fabricate complex geometries. However, the strong sensitivity of NiTi to thermal history and process variability makes predictive modeling and process parameter selection challenging. In this work, a physics-guided decision-support framework is developed for melt pool prediction and stability assessment during the PBF-LB processing of NiTi. A high-fidelity thermal finite element model incorporating CALPHAD-derived, temperature-dependent material properties was calibrated using a subset of experimental measurements and independently validated against additional experimental melt pool data. The calibrated model demonstrated good agreement with experiments, yielding mean absolute percentage errors of 4.22% and 5.63% for melt pool width and depth, respectively, on the validation dataset. A multi-output random forest surrogate trained on the validated simulation dataset enabled rapid prediction of melt pool geometric features, achieving test-set $R^{2}$ values exceeding 0.95, together with low MAE and RMSE values, while five-fold cross-validation confirmed robust predictive performance. The proposed framework integrates surrogate predictions with physics-based melt pool stability criteria to rapidly identify physically feasible processing conditions, thereby providing a computationally efficient foundation for future supervisory process control strategies.

## 1. Introduction

Powder bed fusion–laser beam (PBF-LB) of shape memory alloys such as nickel–titanium (NiTi) enables the fabrication of complex components with tailored thermomechanical properties, including superelasticity and shape memory behavior [1,2,3]. However, these functional properties are highly sensitive to thermal history during processing, as melt pool characteristics directly influence solidification conditions, phase transformation behavior, and defect formation. Variations in laser power, scan speed, beam diameter, and layer thickness can lead to unstable melt pool regimes, resulting in defects such as lack of fusion, keyholing, and balling. Consequently, achieving a stable conduction-mode melt pool is critical for controlling microstructure evolution and ensuring part quality and functional performance [4,5,6].

High-fidelity numerical simulations, particularly thermal models implemented in commercial software such as Ansys Additive Manufacturing Process Parameters (AMPP), provide valuable insight into melt pool evolution and underlying heat transfer mechanisms. These models capture the complex thermal interactions governing melt pool geometry and its influence on process–structure relationships. However, their high computational cost limits their applicability to rapid process exploration, optimization, and real-time decision-making [7,8,9]. As a result, there is growing interest in surrogate modeling approaches that can approximate simulation outputs at a fraction of the computational cost [10,11,12].

Recent studies have applied machine learning techniques to predict melt pool characteristics in laser-based additive manufacturing. While these approaches demonstrate promising predictive accuracy, many remain purely data-driven, offering limited physical interpretability and few safeguards against non-physical predictions [13,14,15]. In the context of manufacturing process control and optimization, this lack of embedded physical knowledge poses a significant challenge, as mathematically optimal solutions may correspond to physically unstable or unmanufacturable regimes, limiting their practical applicability in process design and control [16,17,18].

To address these limitations, physics-informed digital twins have emerged as a powerful paradigm for additive manufacturing. Rather than relying solely on black-box regression, such frameworks integrate data-driven surrogate models with explicit physical constraints, domain knowledge, and decision logic, not only to predict process outcomes rapidly but also to assess process stability and guide parameter selection in a physically meaningful manner. This capability is particularly important for materials such as NiTi, where melt pool stability governs defect formation, thermal gradients, and, ultimately, microstructure-dependent functional properties [19,20,21,22].

In this work, an offline physics-guided decision-support framework is developed for melt pool prediction and stability assessment in the PBF-LB processing of NiTi. A high-fidelity thermal finite element model implemented in Ansys AMPP, calibrated using experimental measurements, is used to generate a comprehensive dataset of melt pool geometries. A multi-output surrogate model is then trained to predict melt pool width, depth, and length with high accuracy. Physics-based stability criteria, formulated using dimensionless geometric ratios, are embedded in the framework to classify processing regimes, including conduction, keyhole, balling, and lack of fusion.

The proposed framework further functions as a decision engine by enabling physics-constrained optimization of laser process parameters toward stable conduction-mode operation. By combining rapid surrogate evaluation with rule-based physical reasoning, the framework provides both quantitative predictions and qualitative insight into solidified melt pool geometry. The present implementation represents a process-level digital twin operating in an offline predictive mode. While the framework currently relies on surrogate predictions generated from experimentally calibrated thermal simulations, the architecture is designed to be extensible toward real-time implementation through future integration of in situ sensing, feedback communication, adaptive model updating, and closed-loop process control.

## 2. Methodology

### 2.1. Experimental Details

Single-track experiments were conducted on a NiTi substrate using an Aconity MINI metal additive manufacturing system (Aconity GmbH, Herzogenrath, Germany). A layer of gas-atomized, prealloyed NiTi powder was deposited onto the base plate prior to laser exposure. The system was equipped with a laser operating at a wavelength of 1070 nm and a maximum output power of 200 W. The pre-alloyed powder composition consisted of 50.54 at.% nickel (Ni) and 49.46 at.% titanium (Ti), with particle sizes ranging from 15–53 μm. Argon was supplied to the build chamber as a shielding gas to suppress oxidation and maintain a low-oxygen processing environment.

The NiTi base plate had dimensions of 95 × 95 × 6 mm. Throughout the experiments, a solidified powder layer thickness of approximately 30 μm, measured from the multi-beads formed alongside the single tracks, was maintained. To achieve this, an actual powder layer thickness of approximately 150 μm was applied, resulting in a solidified layer thickness of approximately 30 μm and indicating an effective powder packing density of about 20%. The multi-beads were fabricated using constant parameters of 200 W laser power, 1000 mm/s scan speed, and 120 μm hatch spacing. For the single-track experiments, a factorial design of experiments was implemented using a 6 × 6 parameter matrix, in which laser power was varied between 150 and 200 W and scan speed ranged from 500 to 1000 mm/s. The laser beam diameter (spot size at the focal plane) was held constant at 80 μm for all experiments. In total, 36 single-track specimens were fabricated using distinct combinations of laser power and scan speed.

**Figure 1:**
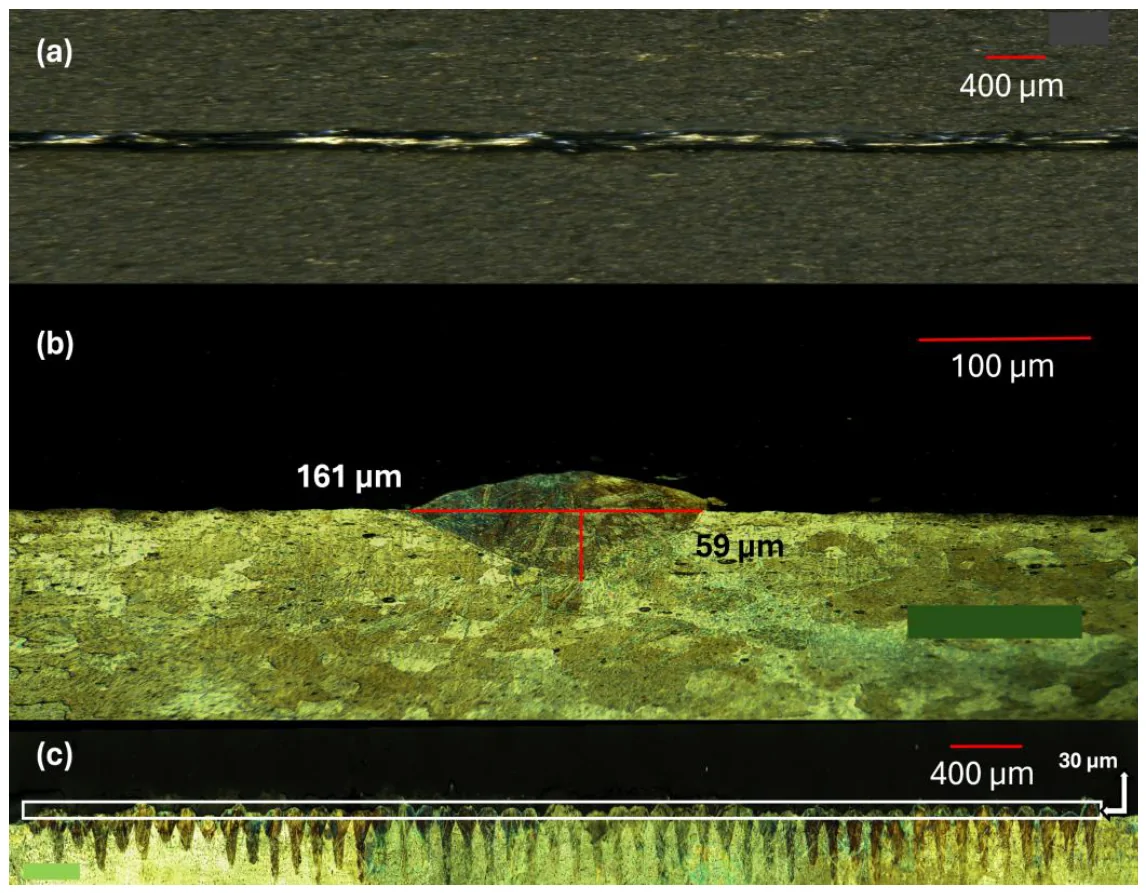
(**a**) Top-view image of a single-track; (**b**) cross-sectional image of a single-track showing melt pool width and depth measurements (Sample 32); (**c**) measurement of the solidified powder layer thickness from the multi-bead experiments.

**Figure A1:**
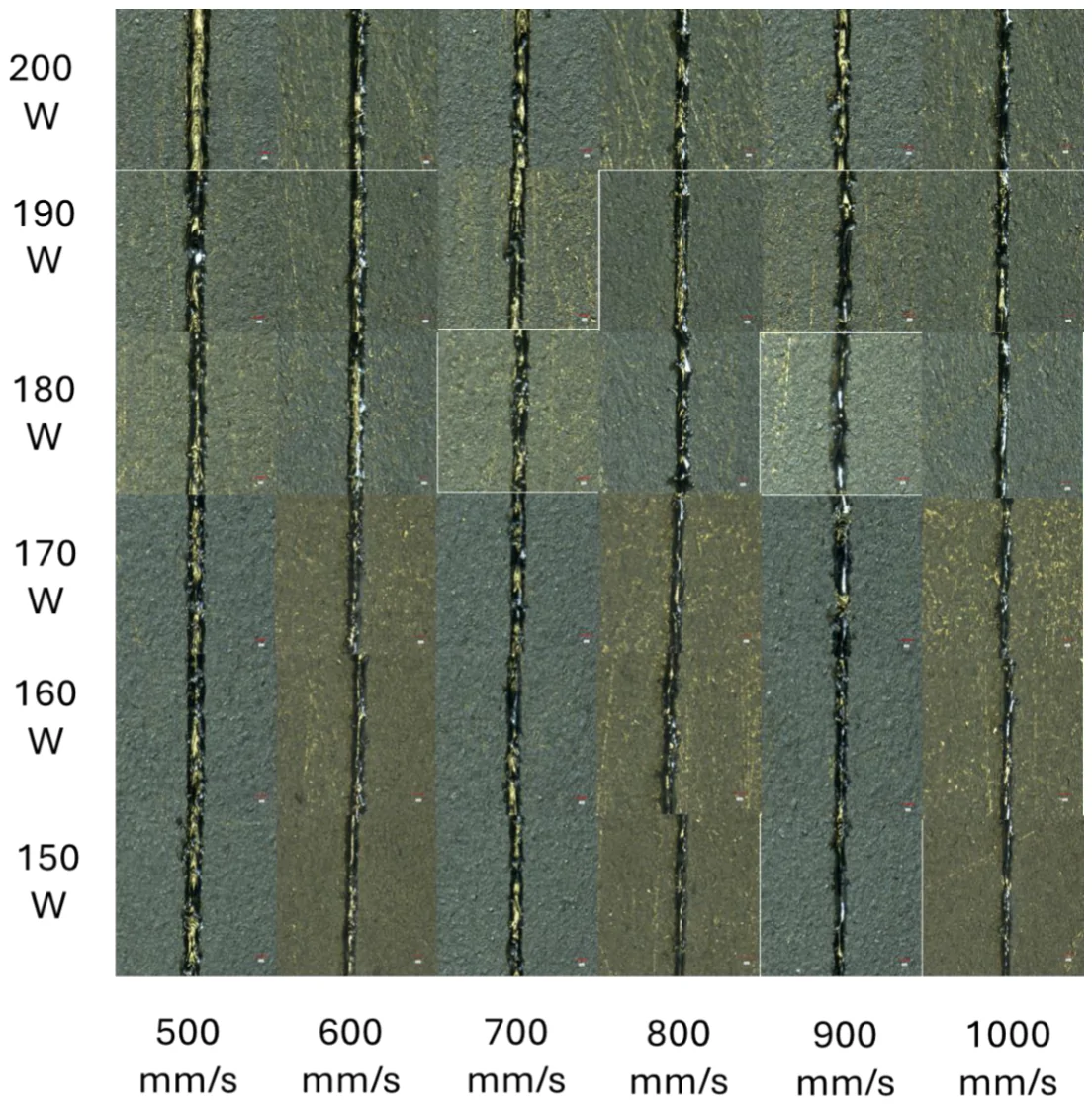
Top-view of the single tracks produced along with the parameter combinations.

**Figure A2:**
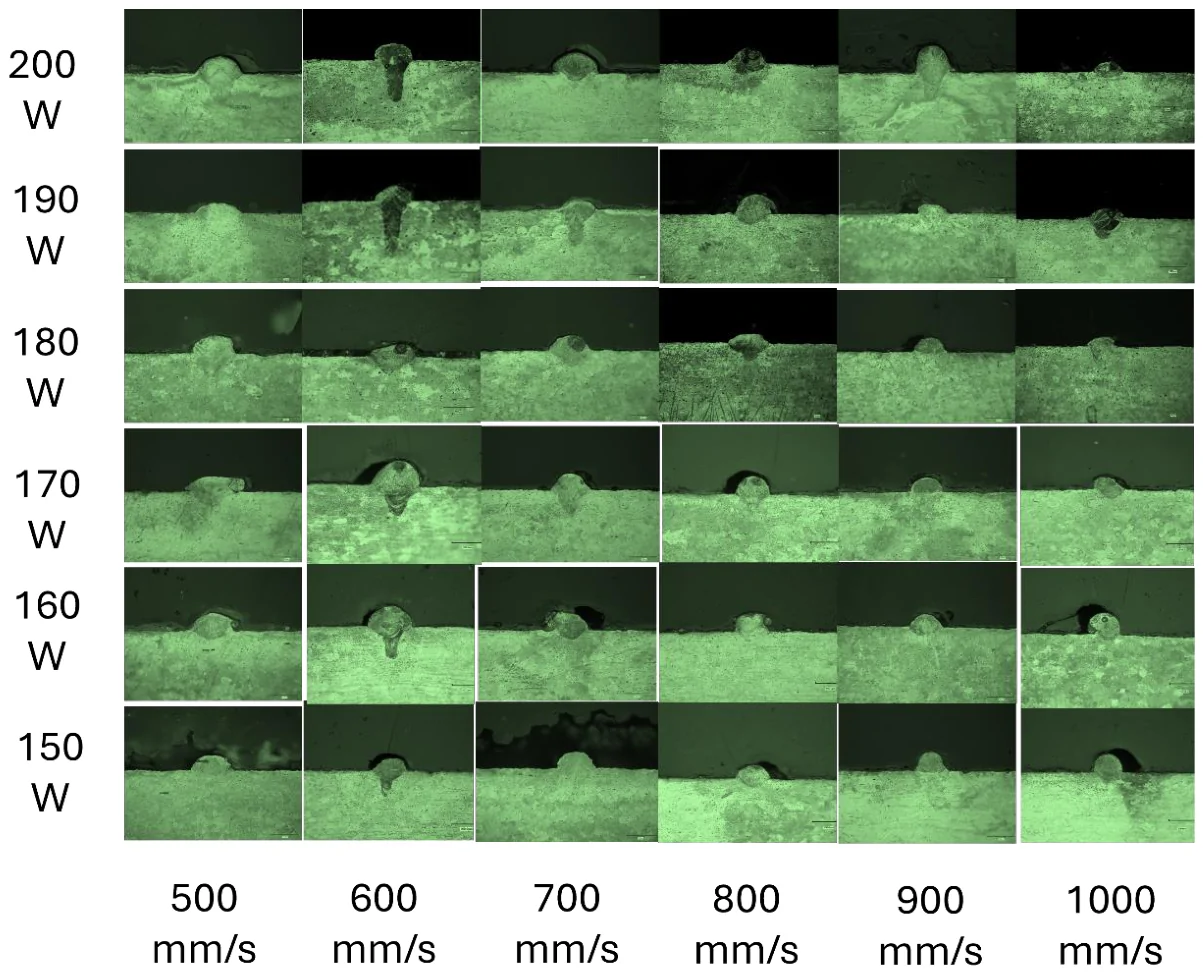
Cross-sectional images of the single tracks produced along with the parameter combinations.

**Figure A3:**
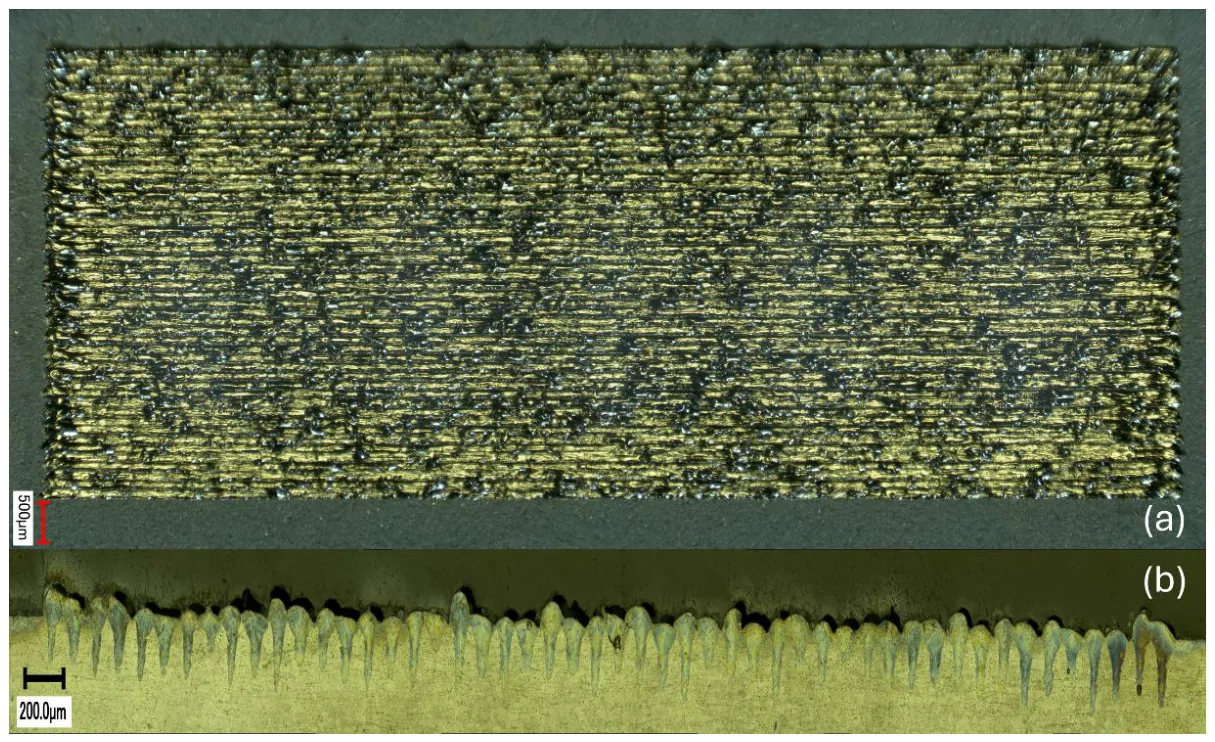
Micrographs of a multi-bead specimen: (**a**) top-view; (**b**) cross-sectional view.

Melt pool widths and depths were quantified using a Keyence VHX-1 three-dimensional optical microscope (Keyence Corporation, Osaka, Japan). Representative images are shown in Figure 1. For cross-sectional characterization, the tracks were sectioned using an ATM Brilliant 250 cutting machine approximately at the center of each track (ATM Qness GmbH, Mammelzen, Germany). The extracted sections were mounted in a transparent hot mounting resin and prepared using standard metallographic procedures. Grinding was performed with silicon carbide papers of progressively finer grit sizes from 80 to 1200, followed by polishing with diamond suspensions of 9, 3, and 1 μm particle size at rotational speeds between 250 and 300 rpm. To reveal melt pool morphology, the polished samples were chemically etched using a solution containing 5 mL nitric acid (HNO_3_), 4 mL hydrofluoric acid (HF), and 40 mL deionized (DI) water. Additional top-view and cross-sectional images of the fabricated tracks are provided in Appendix A, Figure A1, Figure A2 and Figure A3.

### 2.2. Pyrometric Infrared (IR) Data

Thermal measurements of the molten pool were obtained using two pyrometers manufactured by KLEIBER Infrared GmbH (Unterwellenborn, Germany), integrated into the Aconity MINI PBF-LB system (Aconity GmbH, Herzogenrath, Germany). These sensors capture emitted radiation from the laser–material interaction zone within a wavelength range of 1500–1700 nm. The acquired infrared (IR) data are stored along with their corresponding spatial coordinates (x, y) on the build plate.

In this work, the recorded data were processed to generate graphical visualizations of temperature variations across the build layer. The data acquisition rate was 100,000 samples per second, resulting in about 1,065,000 data points for a single layer, each containing spatial and thermal information. To facilitate comparison and visualization of the thermal behavior, the temperature values were normalized to a dimensionless scale between 0 and 1.

### 2.3. CALPHAD

**Figure 2:**
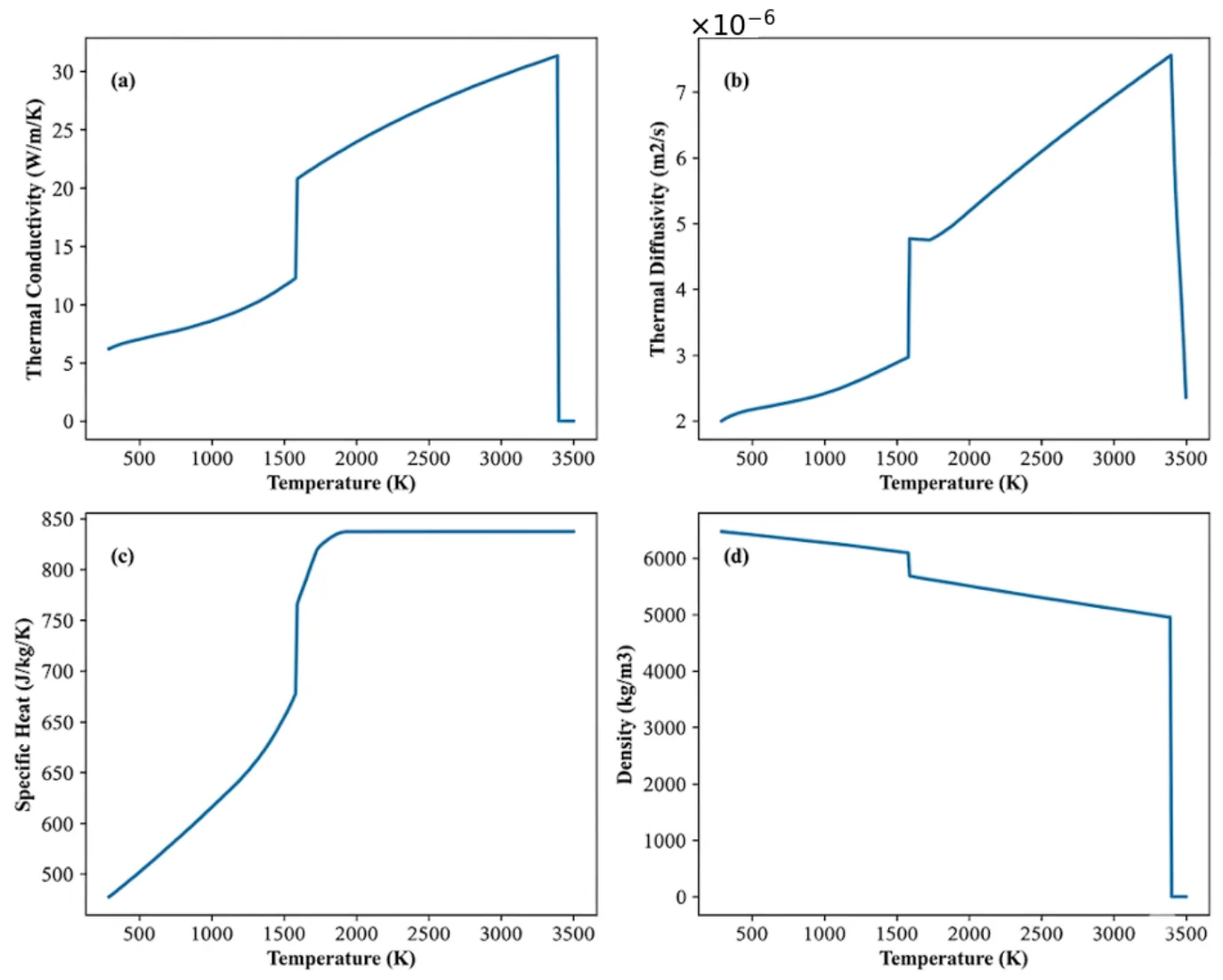
CALPHAD (**a**) Thermal conductivity, (**b**) thermal diffusivity, (**c**) specific heat, and (**d**) density of NiTi as functions of temperature.

The temperature-dependent NiTi material properties were obtained using the CALPHAD method in Thermo-Calc 2025a software, employing the TCTI6 titanium alloys database and the Equilibrium with Freeze-in Temperature model. This titanium-alloys database is the most up-to-date database for shape memory alloys, as it contains both the B2 austenite phase and the B19′ martensite phase. These properties, namely thermal conductivity, diffusivity, specific heat capacity, and density, are shown in Figure 2.

### 2.4. Thermal Modelling

**Figure 3:**
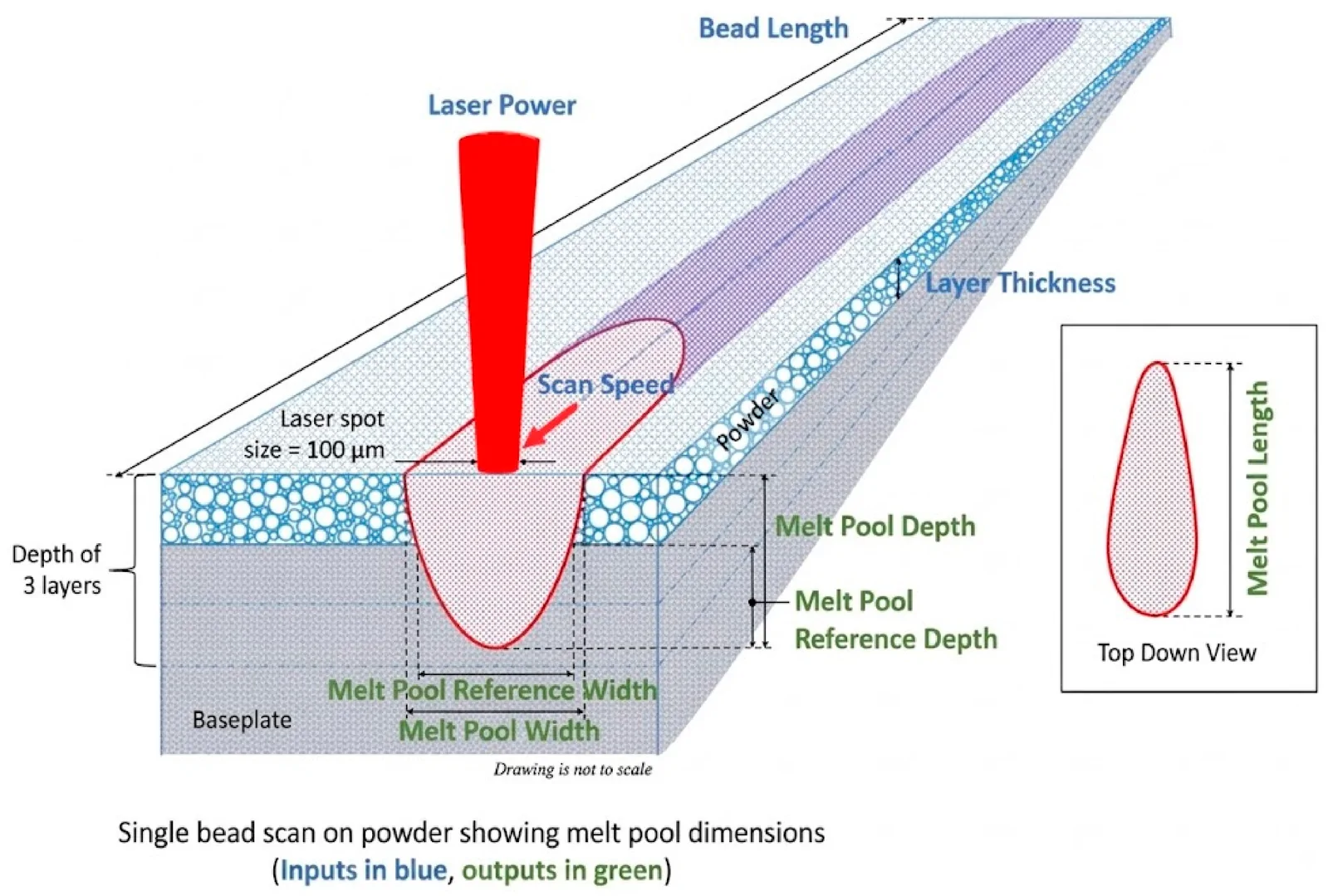
Illustration of the baseplate, single-bead, and their dimensions in the model, adapted from the ANSYS Additive User’s Guide (2020).

A three-dimensional transient thermal finite element (FE) model was developed in Ansys Additive Manufacturing Process Parameters (AMPP) 2025 R2 to simulate single-track melting during PBF-LB, as illustrated in Figure 3. The simulations employed a structured Cartesian computational grid to maintain fixed spatial coordinates and facilitate accurate extraction of melt pool geometric features. A uniform in-plane mesh resolution of 15 μm was adopted in the X–Y directions, while the mesh spacing in the build (Z) direction was automatically adjusted by AMPP according to the ratio of the layer thickness to the prescribed number of mesh layers, resulting in a comparable spatial resolution in all three directions. This discretization was internally verified by Ansys through mesh-convergence studies and was considered sufficient for accurately resolving melt pool geometries within the investigated dimensional range.

NiTi was implemented as a user-defined material using the temperature-dependent thermophysical properties obtained from CALPHAD calculations (Figure 2). The thermal simulations were performed using the material properties, boundary conditions, and numerical settings summarized in Table 1. Melting and solidification were represented using the enthalpy-based phase-change formulation implemented in Ansys AMPP, while the Gaussian heat-source parameters governing laser absorptivity and penetration depth were calibrated using the experimental dataset. All other thermophysical material properties were retained unchanged throughout the calibration process.

Approximately one quarter of the experimental dataset (Samples 10–18 in Table 2) was used to calibrate the thermal model. Calibration was performed by tuning the Gaussian heat-source parameters governing laser absorptivity and penetration depth, while all CALPHAD-derived thermophysical properties were retained unchanged. The remaining experimental conditions (Samples 1–9 and 19–36 in Table 2) were reserved exclusively for independent validation of the calibrated model. The governing equations of the thermal model are presented in Equations (1)–(3), where Equation (1) describes transient heat conduction based on Fourier’s law, Equation (2) represents convective heat loss from the exposed surfaces, and Equation (3) defines the Gaussian laser heat source.

(1) $$\rho c\frac{\partial T}{\partial t}=\frac{\partial }{\partial x}\left(k\frac{\partial T}{\partial x}\right)+\frac{\partial }{\partial y}\left(k\frac{\partial T}{\partial y}\right)+\frac{\partial }{\partial z}\left(k\frac{\partial T}{\partial z}\right)+Q$$

where *ρ* is density, *c* is specific heat, *k* is thermal conductivity, *T* is temperature, *t* is time, *Q* is heat source, and *x*, *y*, and *z* denote the spatial directions.

(2) $$Q_{c}=h(T-T_{0})$$

where *Q_c_* is the heat loss due to convection, *h* is the coefficient of thermal convection, *T* is transient temperature, and *T*_0_ is the initial temperature.

(3) $$Q=2\frac{AP}{\pi w^{2}}\exp\frac{-2r^{2}}{w^{2}}$$

where *Q* is the heat flux input, *A* is the absorptivity, *P* is the laser power, *w* is the laser beam diameter, and *r* is the position of the laser beam.

A complete list of symbols and abbreviations used throughout the manuscript is provided in Appendix A, Table A1 and Table A2.

In the thermal model, temperature-dependent ratios between solid and powder properties are applied to account for the lower effective conductivity and density of the powder. This approach allows the model to represent not only the room-temperature powder behavior but also the thermal properties at elevated temperatures prior to melting. Details of the ratios between the powder and solid properties are given below:

- Thermal Conductivity Ratio values:
  ○ Ratio = 0.01 from 2 Kelvin to [0.6 × the solidus temperature].
  ○ Ratio = 0.4 from [0.6 × the solidus temperature] to the liquidus temperature of the material.
  ○ Ratio = 1 from the liquidus temperature to 15,000 Kelvin.
- Density Ratio values:
  ○ Ratio = 0.2 from 2 Kelvin to the liquidus temperature of the material.
    (0.2 is the value that corresponds to the powder packing density in the build chamber after the recoating process)
  ○ Ratio = 1 from the liquidus temperature to 15,000 Kelvin.
- Specific Heat Ratio value is always 1.

Other boundary conditions in the model are:

- The top boundary of the build has a constant forced convection applied uniformly. Aside from the laser model, radiation effects are neglected.
- The lateral boundary conditions are adiabatic, while a buffer region of powder extends beyond the part’s bounding box to avoid boundary effects.
- The user-specified baseplate temperature is used as a fixed-temperature boundary condition at the bottom of the build.

For further details of the model, the reader is referred to the work by Teng et al. [23].

### 2.5. Machine Learning

#### 2.5.1. Data Generation and Pre-Processing

Thermal simulation data were generated using the AMPP thermal model for single-track laser melting. The dataset comprises process parameters and corresponding melt pool geometric characteristics extracted from the simulated transient thermal fields. All data were assembled into a structured dataset and used directly without normalization, as tree-based models are invariant to monotonic feature scaling.

#### 2.5.2. Surrogate Model Development

A data-driven surrogate model was developed to approximate the mapping between laser process parameters and melt pool geometry obtained from high-fidelity AMPP thermal simulations. A random forest regressor was selected due to its suitability for limited datasets, ability to capture nonlinear interactions between process parameters, and improved robustness through ensemble averaging. To mitigate overfitting, model complexity was controlled using constrained tree depth and minimum leaf size parameters.

The final surrogate model employed 400 decision trees (n_estimators = 400), with a maximum tree depth of 18 and a minimum leaf size of 2. These parameters were selected empirically to balance predictive accuracy and model generalization. To predict multiple geometric outputs simultaneously, a multi-output regression framework was implemented using a MultiOutputRegressor wrapper.

The thermal simulations were performed on a workstation equipped with an Intel Xeon W-2275 CPU (3.30 GHz), 128 GB RAM, and 14 cores. Each simulation required approximately 15 min to compute the melt pool geometry for a single set of process parameters. In contrast, the developed surrogate-based decision-support system enables prediction of melt pool characteristics in less than one second. This represents a reduction in computational time by over three orders of magnitude, highlighting the potential of the proposed framework for rapid process exploration, optimization, and real-time decision support in the PBF-LB processing of NiTi.

#### 2.5.3. Physics-Guided Offline Digital Twin Architecture

The trained surrogate model was integrated into a physics-guided decision-support framework for rapid prediction and assessment of melt pool behavior in the PBF-LB processing of NiTi. The framework combines data-driven surrogate predictions with explicit physics-based regime classification and constrained decision logic to support process exploration within physically meaningful operating conditions. Unlike conventional physics-informed machine learning approaches, physical constraints are not embedded directly into the surrogate model training objective. Instead, physically motivated melt pool stability criteria are incorporated at the decision-engine level to guide parameter selection and regime assessment rather than being directly embedded as governing equations within the surrogate model loss function.

The system maps a vector of PBF-LB process parameters (Equation (4)):

(4) $$x=\{h,d_{b},P,v\}$$

where

- h = layer thickness (µm)
- $d_{b}$ = beam diameter (µm)
- P = laser power (W)
- v = scan speed (mm/s)

To predicted melt pool geometric features (Equation (5)):

(5) $$y=\{W,D,W_{ref},D_{ref},L\}$$

where

- W = melt pool width
- D = melt pool depth
- $W_{ref}$ = melt pool reference width
- $D_{ref}$ = melt pool reference depth
- L = melt pool length

Melt pool width corresponds to the width measured at the top surface of the deposited powder layer, while melt pool depth represents the combined depth of the remelted region and the powder layer thickness. Reference melt pool width and reference melt pool depth are defined at the top of the remelted substrate beneath the deposited powder layer and therefore characterize only the remelted substrate geometry.

The surrogate model $\hat{f}(\cdot )$ was trained to approximate the calibrated AMPP thermal simulation response (Equation (6)):

(6) $$\hat{y}=\hat{f}(x)$$

The surrogate model consists of a random forest regressor implemented in a multi-output framework (Equation (7)):

(7) $$\hat{f}(x)=\left[\hat{W}(x),\hat{D}(x),{\hat{W}}_{ref}(x),{\hat{D}}_{ref}(x),\hat{L}(x)\right]$$

To improve generalization assessment, the surrogate model was evaluated using an 80/20 train-test split together with five-fold cross-validation. The final deployed surrogate was subsequently retrained using the full dataset to maximize predictive fidelity within the calibrated process space.

#### 2.5.4. Decision Engine and Physics-Constrained Parameter Search

The framework was coupled with a physics-constrained decision engine to identify process parameter combinations associated with stable conduction-mode melting. Rather than performing gradient-based optimization, the framework employs a surrogate-assisted discrete parameter search constrained to the validated design-of-experiments (DOE) parameter space. The search space was defined using laser power and scan speed as optimization variables while maintaining fixed layer thickness and beam diameter (Equation (8)).

(8) z={P,v}

For fixed layer thickness and beam diameter, the decision engine performs a discrete parameter search over predefined laser power and scan speed combinations corresponding to the validated DOE space used for surrogate model training. Restricting the search to DOE-consistent parameter combinations prevents extrapolation beyond the calibrated surrogate domain and improves the physical reliability of the predictions. Candidate parameter combinations were evaluated using the surrogate model together with physics-based regime classification criteria.

A candidate parameter set was considered feasible only if it satisfied the conduction-regime conditions (Equation (9)). Among the feasible candidates, a scalarized objective function was used to quantify melt pool stability, penalizing deviations from the target geometric ratios:

- Reference depth-to-width ratio near 0.5
- Length-to-width ratio between 2 and 3

(9) F={z∣Regime(z)=Conduction}

A scalarized stability objective was minimized (Equation (10)):

(10) $$J(z)=|R_{DW}(z)-0.5|+|R_{LW}(z)-2.5|$$

Regime classification was determined using dimensionless geometric ratios derived from the predicted melt pool dimensions:

(11) $$R_{DW}=\frac{D_{ref}}{W_{ref}}$$

(12) $$R_{LW}=\frac{L}{W}$$

where $R_{DW}$ represents the reference depth-to-width ratio and $R_{LW}$ denotes the melt pool length-to-width ratio. The target values of 0.5 and 2.5 correspond to characteristic geometric ratios associated with stable conduction-mode melting reported in the literature. Minimization of J(z) therefore selects parameter combinations closest to the desired stable melt pool morphology while remaining within the feasible conduction regime. The first term penalizes deviation from characteristic conduction-mode penetration behavior, while the second term penalizes excessive melt pool elongation associated with balling instability. The candidate minimizing this stability metric was selected as the optimal solution (Equation (13)). The optimal parameters were selected as follows:

(13) $$z^{*}=arg\;\underset{z\in F}{min}\;J(z)$$

The proposed framework employs a surrogate-assisted parameter exploration strategy rather than a formal gradient-based optimization algorithm. Candidate process parameters are sampled within predefined physically valid ranges and evaluated using the surrogate model. Predicted melt pool geometries are subsequently filtered using physics-based regime classification criteria to identify feasible conduction-mode conditions.

The regime classification follows a hierarchical, physics-guided, rule-based decision sequence calibrated against the experimentally observed melt-pool morphologies. Lack of fusion is evaluated first because insufficient penetration represents a fundamental process failure that overrides all subsequent criteria. Keyhole formation is then assessed based on excessive penetration. Stable conduction mode is subsequently assigned when the reference depth-to-width ratio lies between 0.30 and 0.65, the reference depth is at least equal to the layer thickness, and the melt-pool length-to-width ratio does not exceed 4. Processing conditions that do not satisfy the criteria for lack of fusion, keyhole, or stable conduction are classified as transitional.

A scalarized stability objective function is then evaluated for all feasible candidates, and the parameter combination minimizing the deviation from the target geometric stability ratios is selected as the preferred operating condition.

#### 2.5.5. Graphical User Interface Integration

**Figure A4:**
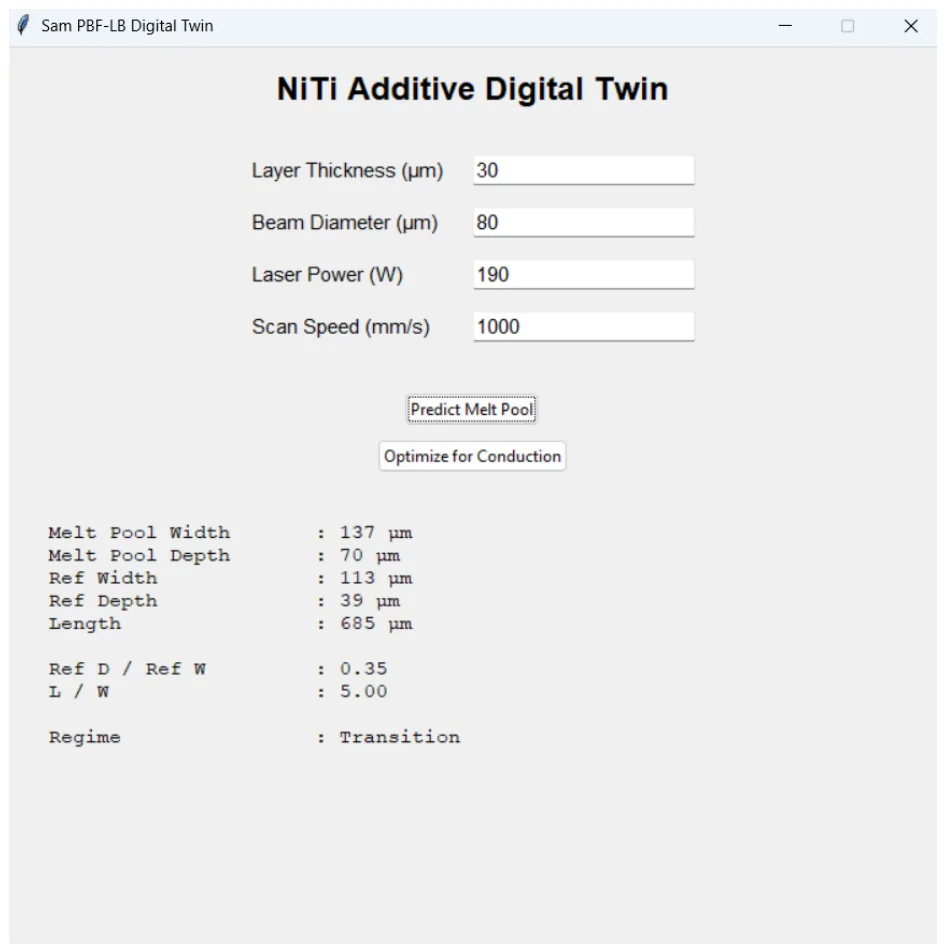
GUI showing melt pool prediction.

**Figure A5:**
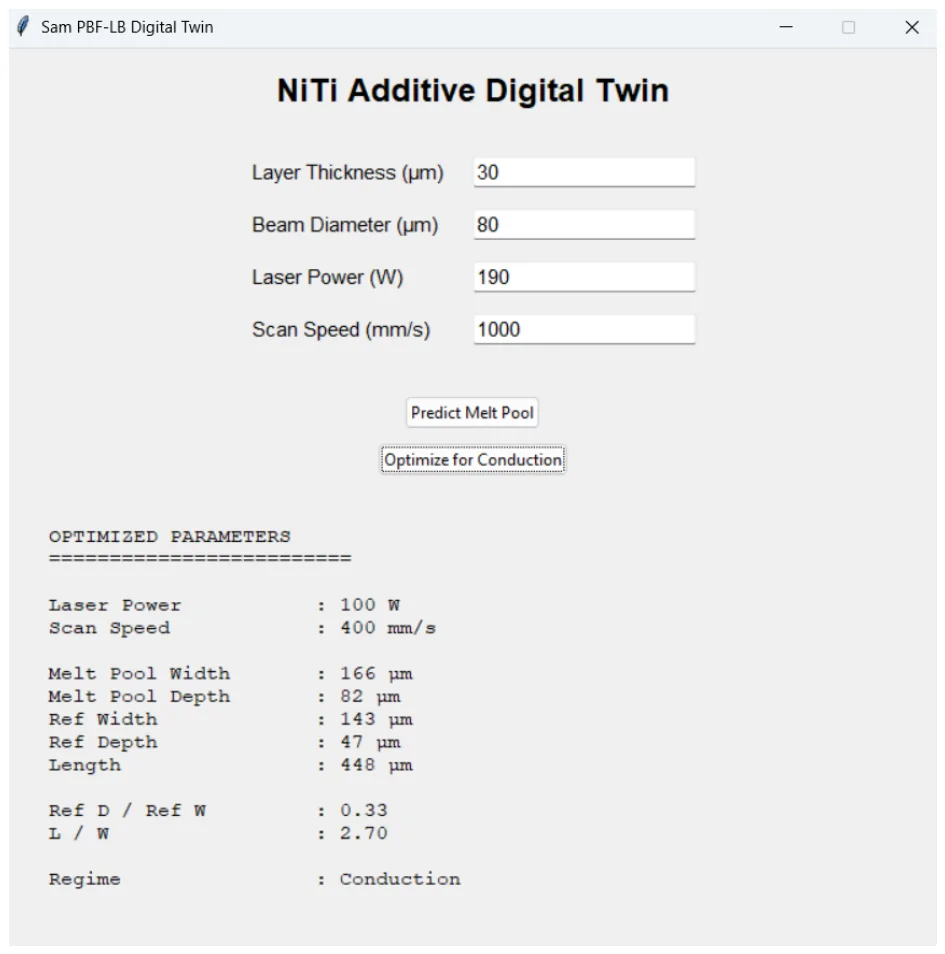
GUI showing process optimization.

A graphical user interface (GUI) was developed using the Tkinter framework to enable interactive use of the decision-support system. The GUI (shown in Appendix A, Figure A4 and Figure A5) allows users to:

- Enter PBF-LB process parameters;
- Predict melt pool geometry and processing regime; and
- Perform physics-constrained parameter search for conduction-mode operation.

**Figure 4:**
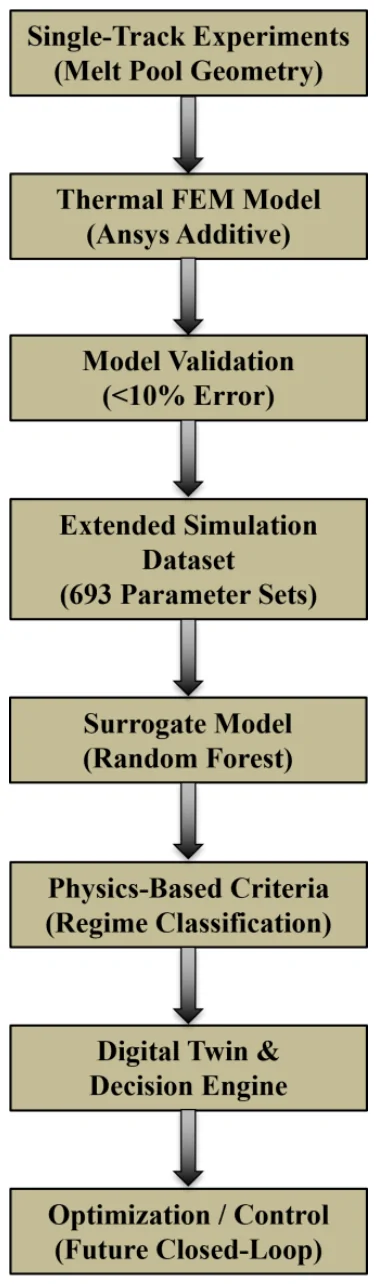
Workflow schematic of the physics-guided decision-support system.

The interface displays predicted melt pool dimensions, geometric stability ratios, and the corresponding melt pool regime classification in real time, thereby enabling rapid engineering assessment without direct interaction with computationally intensive thermal simulations. Figure 4 illustrates the overall workflow of the proposed framework.

## 3. Results and Discussion

### 3.1. IR Data Analysis

**Figure 5:**
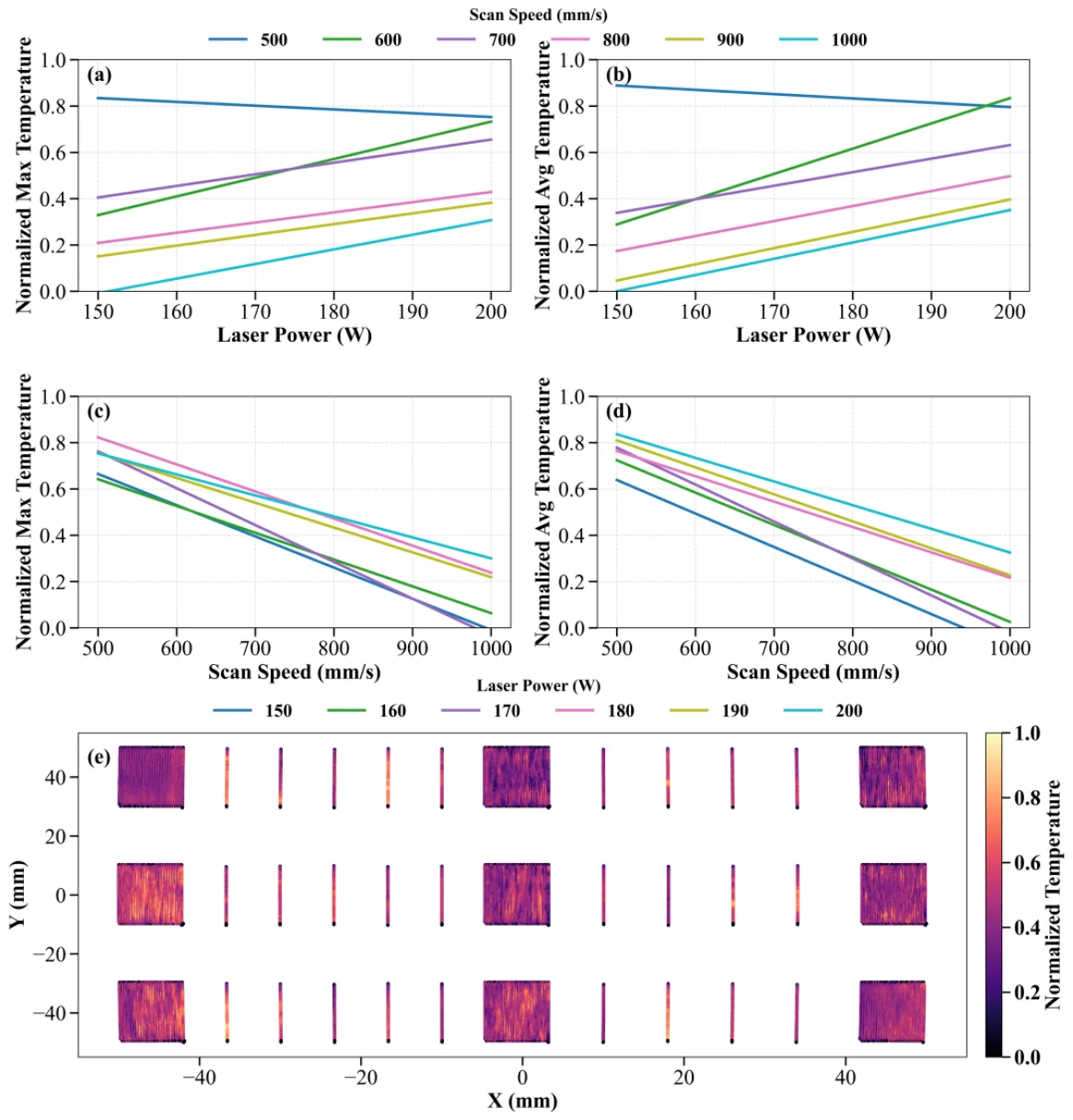
Effects of laser power on (**a**) maximum temperature and (**b**) average temperature; effects of scan speed on (**c**) maximum temperature and (**d**) average temperature; and (**e**) thermogram visualization of single tracks and multi-beads together with the corresponding temperatures recorded by pyrometric IR data monitoring.

Figure 5 shows the relationship among laser power, scan speed, and temperature recorded by the pyrometers, as well as the thermogram layout of the printed single-tracks and multi-beads. It can be observed that the temperature increases with increasing laser power and decreases with increasing scan speed. This trend reflects the effect of energy density on melt pool temperature and, consequently, on the melt pool dimensions, with an increase in energy density leading to increases in these attributes and vice versa [24].

### 3.2. Experimental Melt Pool Trend Analysis

**Figure 6:**
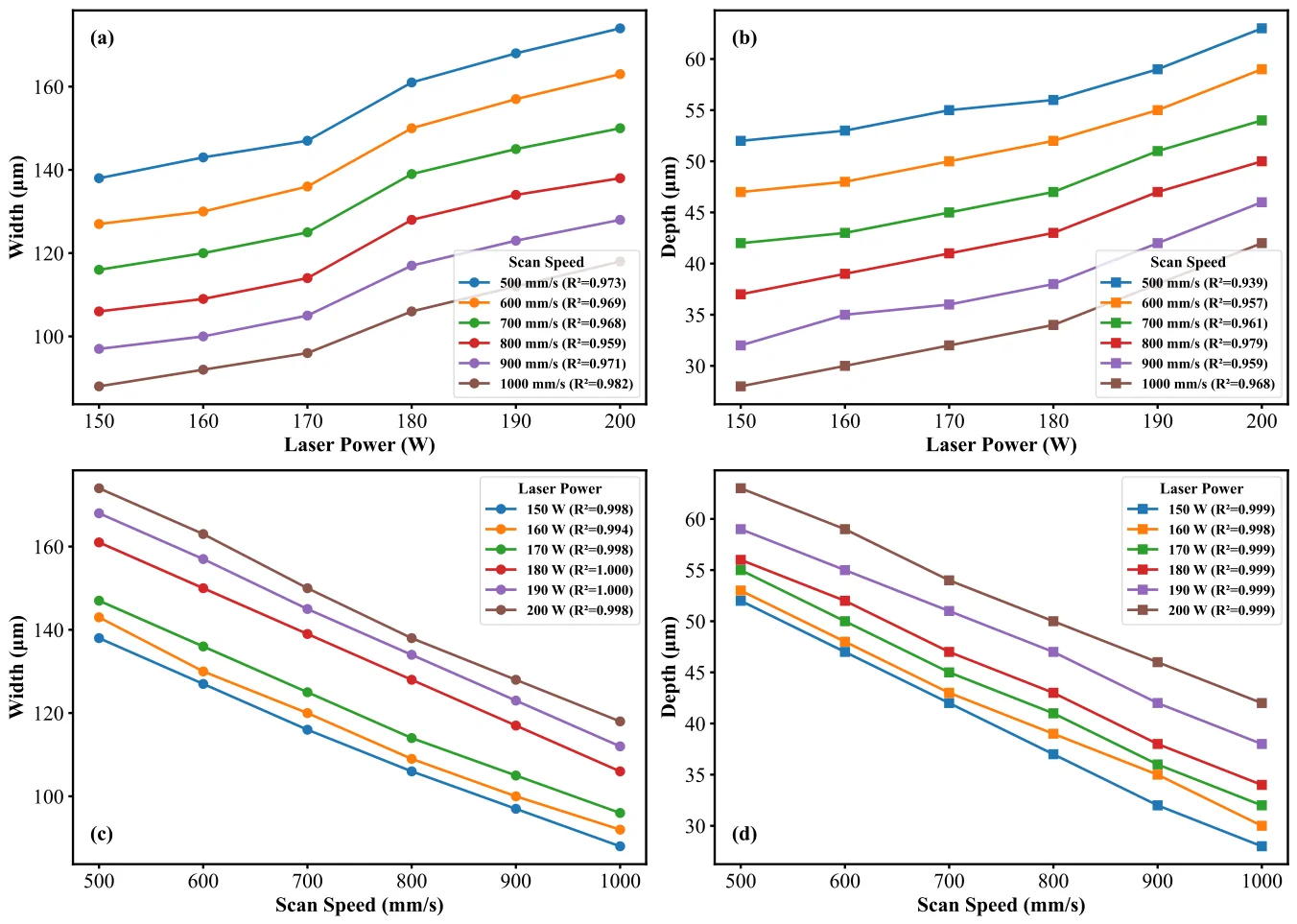
(**a**) Effect of laser power on melt pool width, (**b**) effect of laser power on melt pool depth; (**c**) effect of scan speed on melt pool width; and (**d**) effect of scan speed on melt pool depth, based on the experimental measurements, together with the corresponding R^2^ values.

Figure 6 presents the experimentally measured variation in melt pool width and depth as functions of laser power and scan speed under different processing conditions. The experimental results reveal clear and physically consistent trends, with melt pool width and depth increase systematically with increasing laser power, whereas both quantities decrease with increasing scan speed, consistent with the trends observed in the IR melt pool temperature data (Figure 5). At higher laser powers, the increased thermal energy input promotes greater heat accumulation and enhanced melting, resulting in wider and deeper melt pools. Conversely, increasing scan speed reduces the interaction time between the laser and the material, thereby decreasing the absorbed energy per unit length and limiting melt pool growth. The observed behavior agrees well with established thermophysical interpretations reported in the literature for laser-based additive manufacturing processes [25,26,27].

### 3.3. Thermal Model Performance

**Figure 7:**
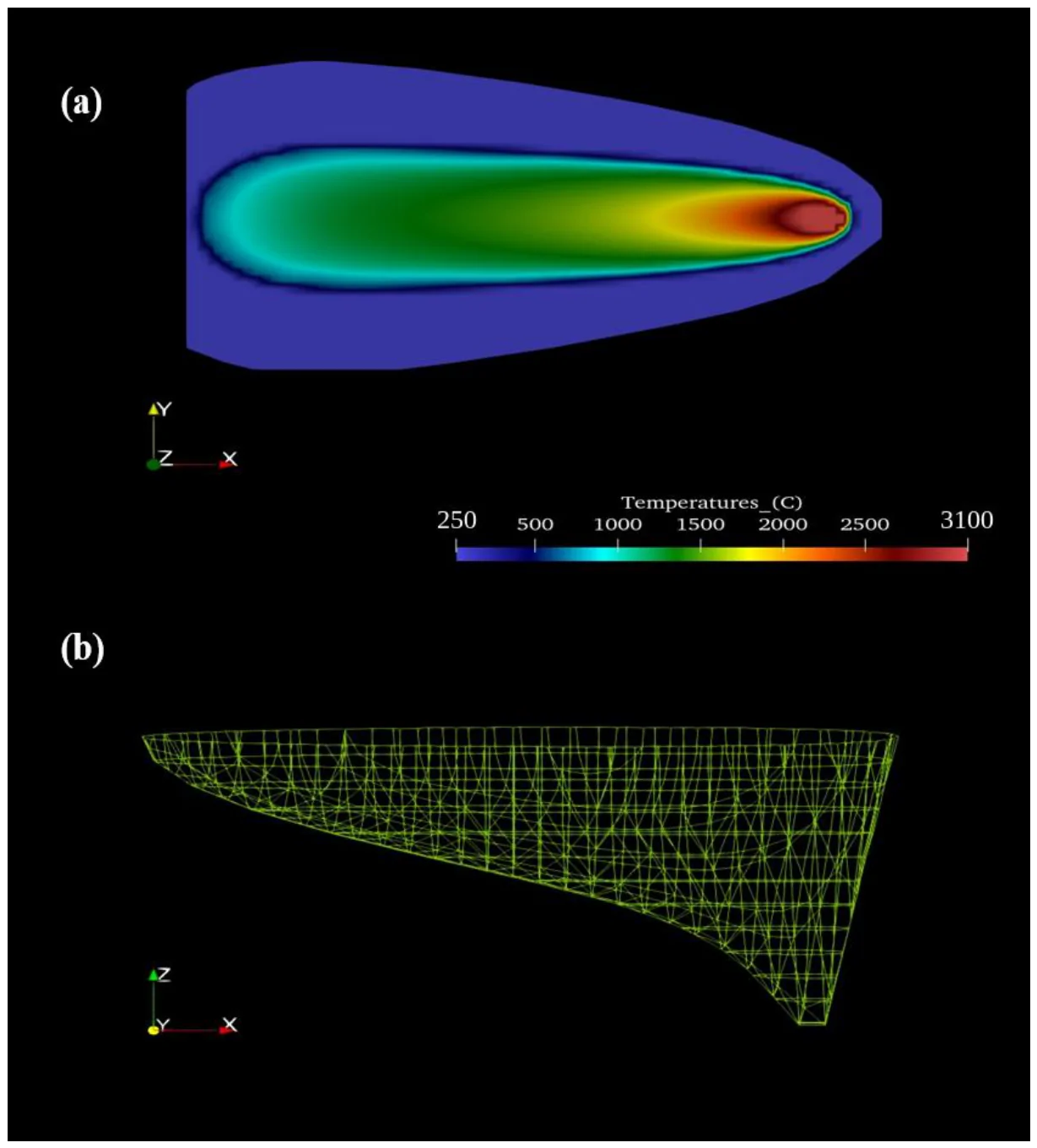
Simulated melt pool obtained from the thermal model for parameters 200 W laser power, 500 mm/s scan speed, 30 μm layer thickness, 80 μm beam diameter: (**a**) top-view; (**b**) side view.

The calibrated thermal model was subsequently evaluated using an independent validation dataset comprising Samples 1–9 and 19–36, which were not used during model calibration. Following calibration using Samples 10–18, the FE thermal model was applied to simulate melt pool geometries for all 36 experimental process conditions. The predicted melt pool dimensions exhibited good agreement with the experimental measurements across the investigated process window. A detailed comparison between the experimental and simulated melt pool dimensions for each processing condition, along with the corresponding prediction errors, is presented in Table 2, while the corresponding summary statistics for the calibration and independent validation datasets are provided in Table 3. The results demonstrate that the calibrated thermal model accurately reproduces the experimentally observed melt pool behavior and provides a reliable foundation for subsequent surrogate model development. In addition to width and depth, the thermal model was used to estimate melt pool length, which is difficult to measure reliably using the experimental setup. A representative example of a simulated melt pool is shown in Figure 7.

**Figure 8:**
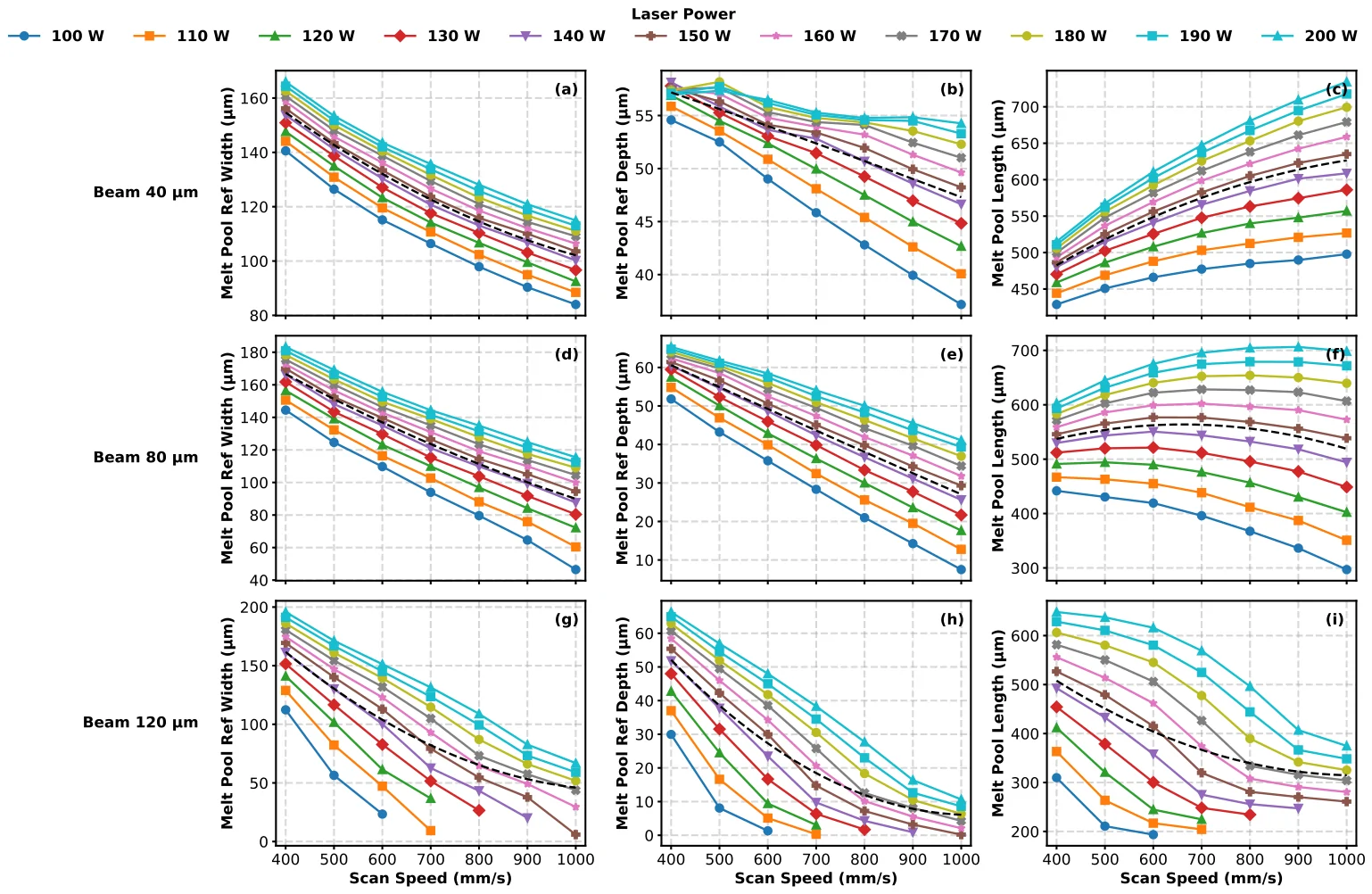
Thermal-model-predicted melt pool dimensions for a 30 µm layer thickness—(**a**) melt pool width, (**b**) melt pool depth, and (**c**) melt pool length for a 40 µm beam diameter; (**d**) melt pool width, (**e**) melt pool depth, and (**f**) melt pool length for 80 µm beam diameter; and (**g**) melt pool width, (**h**) melt pool depth, and (**i**) melt pool length for a 120 µm beam diameter. Dashed lines represent the overall trend.

**Figure 9:**
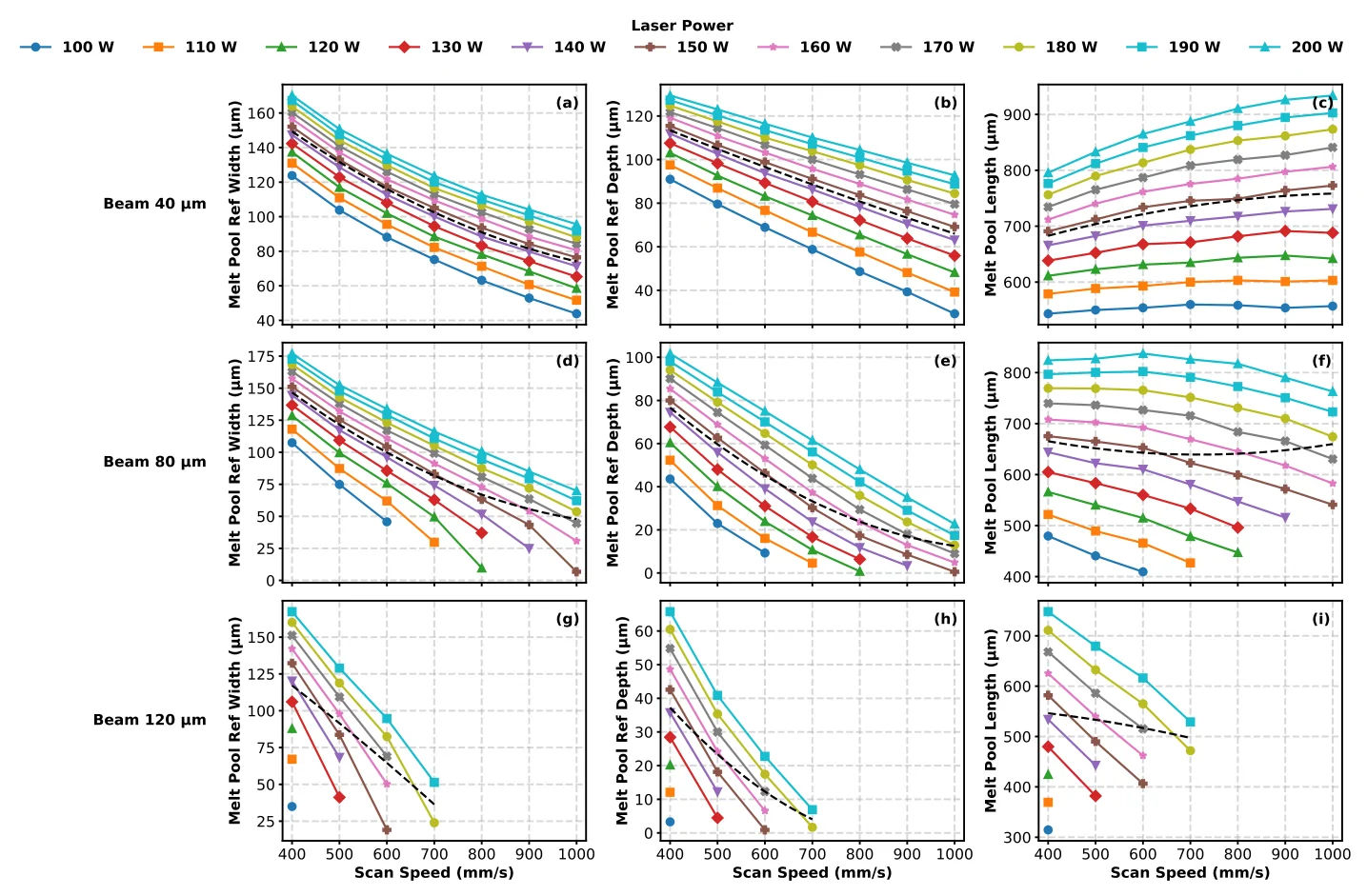
Thermal-model-predicted melt pool dimensions for a 60 µm layer thickness—(**a**) melt pool width, (**b**) melt pool depth, and (**c**) melt pool length for a 40 µm beam diameter; (**d**) melt pool width, (**e**) melt pool depth, and (**f**) melt pool length for an 80 µm beam diameter, and (**g**) melt pool width, (**h**) melt pool depth, and (**i**) melt pool length for a 120 µm beam diameter. Dashed lines represent the overall trend.

**Figure 10:**
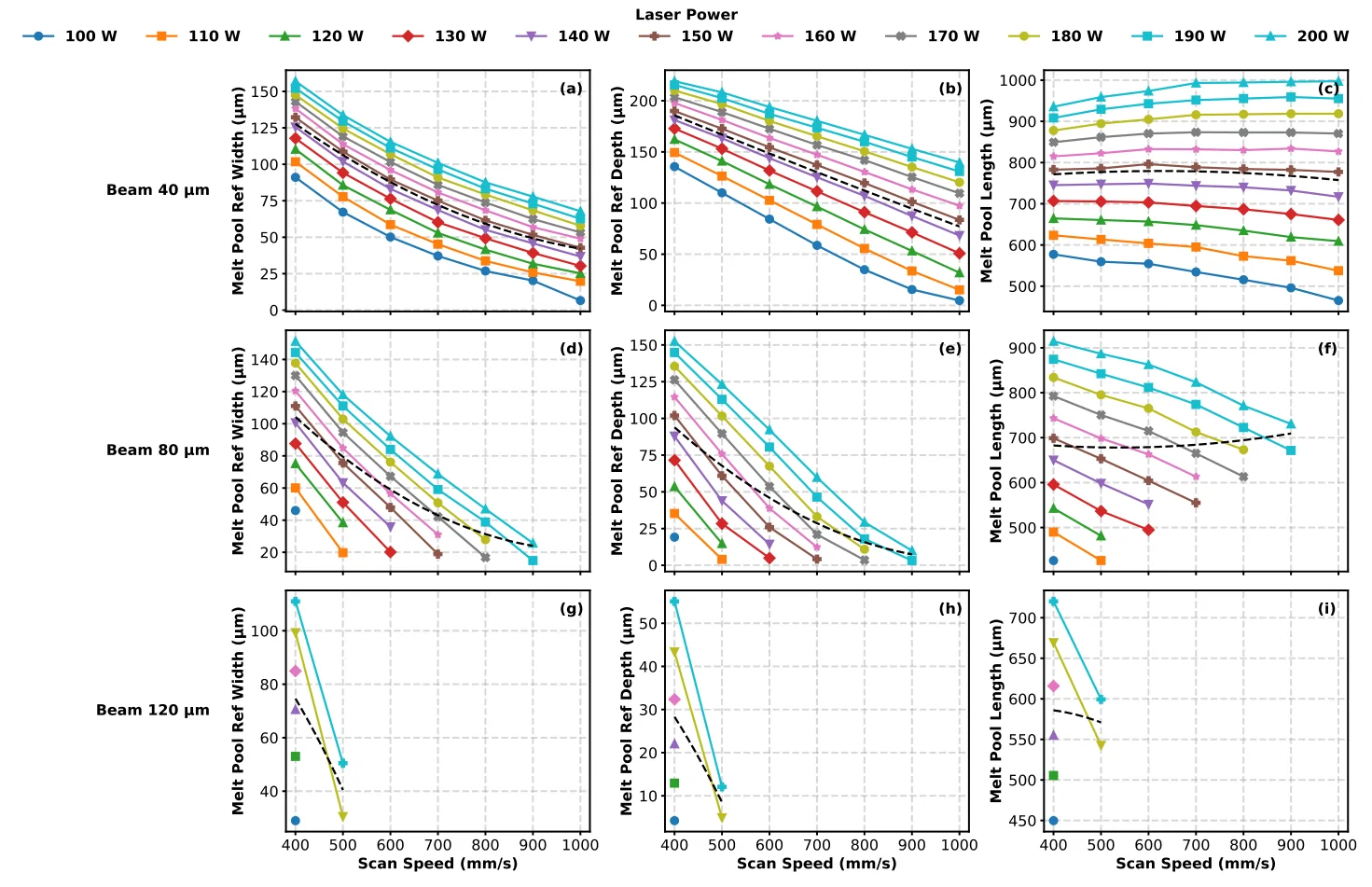
Thermal-model-predicted melt pool dimensions for a 90 µm layer thickness—(**a**) melt pool width, (**b**) melt pool depth, and (**c**) melt pool length for a 40 µm beam diameter; (**d**) melt pool width, (**e**) melt pool depth, and (**f**) melt pool length for an 80 µm beam diameter; and (**g**) melt pool width, (**h**) melt pool depth, and (**i**) melt pool length for a 120 µm beam diameter. Dashed lines represent the overall trend.

Following validation of the thermal model against experimental observations, the dataset was systematically expanded to cover 693 process parameter combinations. This extended dataset comprised three layer thicknesses, three beam diameters, eleven laser power levels, and seven scan speeds. The resulting melt pool dimensions predicted across this expanded parameter space are presented in Figure 8, Figure 9 and Figure 10. For most parameter combinations, the melt pool dimensions exhibit an approximately linear dependence on laser power and scan speed. However, in certain cases, a logarithmic relationship with scan speed is observed. Similar trends were reported in our previous study, where both experimental measurements and predictions from various analytical models demonstrated comparable scan speed–dependent behavior [25]. The comprehensive dataset generated through this approach was subsequently used to train the surrogate model described in the following sections. Although the surrogate model covers a broader range of process parameters than those investigated experimentally, the additional parameter combinations are derived from a calibrated and independently validated thermal model. Consequently, the current framework should be regarded as numerically validated across the full design space, while experimental validation of additional layer thicknesses and beam diameters remains ongoing.

### 3.4. Surrogate Model Performance

The surrogate model performance was evaluated using the coefficient of determination (R^2^), mean absolute error (MAE), and root mean square error (RMSE) on an independent test dataset obtained using an 80/20 train-test split. In addition, five-fold cross-validation was performed to assess model robustness and generalization within the calibrated process parameter space. The multi-output random forest surrogate demonstrated consistently high predictive accuracy across all melt pool geometric descriptors:

- Melt pool width: $R^{2}=0.959$, MAE = 6.19 µm, RMSE = 8.17 µm
- Melt pool depth: $R^{2}=0.991$, MAE = 3.57 µm, RMSE = 5.72 µm
- Reference width: $R^{2}=0.964$, MAE = 6.90 µm, RMSE = 10.42 µm
- Reference depth: $R^{2}=0.982$, MAE = 4.01 µm, RMSE = 6.00 µm
- Melt pool length: $R^{2}=0.983$, MAE = 20.70 µm, RMSE = 28.16 µm

The corresponding five-fold cross-validation results further confirmed model stability, with mean R^2^ values of 0.964, 0.988, 0.962, 0.980, and 0.982 for melt pool width, depth, reference width, reference depth, and melt pool length, respectively. The low standard deviations across folds ($<0.01$) indicate consistent predictive performance and limited sensitivity to data partitioning.

The high $R^{2}$ values demonstrate that the surrogate effectively captures the nonlinear relationships between laser process parameters and melt pool geometry within the simulated process space. The relatively low MAE and RMSE values indicate strong agreement between surrogate predictions and the underlying AMPP thermal simulation outputs in absolute dimensional terms. Among the predicted quantities, melt pool length exhibited comparatively larger errors, which is attributed to its stronger sensitivity to trailing thermal gradients, heat accumulation, and solidification dynamics compared with width- and depth-related features. Overall, the surrogate model provides an accurate and computationally efficient approximation of the calibrated thermal simulations, enabling rapid melt pool prediction suitable for digital twin deployment and physics-constrained parameter exploration.

Because the surrogate model is constructed to emulate the validated FE model, its predictive accuracy with respect to experimental observations is fundamentally bounded by the accuracy of the underlying thermal simulations. Consequently, the experimental validation presented in Table 2 and Table 3 provides the physical basis for the overall prediction framework, while the surrogate validation demonstrates faithful reproduction of the FE model.

### 3.5. Physics-Based Melt Pool Regime Classification

Beyond numerical prediction accuracy, the system incorporates a physics-informed, rule-based framework to classify melt pool processing regimes. Rather than relying solely on surrogate model predictions, the classification combines predicted melt pool geometry with experimentally calibrated morphological criteria derived from the 36 validated single-track melt pool cross-sections. This approach integrates a physical understanding of melt pool formation with experimentally observed transitions between stable and unstable melting conditions.

Regime classification is based on two dimensionless geometric descriptors derived from the predicted melt pool geometry: the reference depth-to-width ratio $\left(D_{ref}/W_{ref}\right)$ and the melt pool length-to-width ratio $\left(L/W\right)$. These descriptors quantify the balance among laser penetration, lateral heat conduction, and melt pool morphology, enabling the physically meaningful identification of process stability.

The following regimes are identified within the framework:

- Lack of fusion, corresponding to insufficient remelting when the predicted reference melt pool depth is less than approximately 85% of the deposited layer thickness, indicating inadequate penetration for complete layer consolidation [28].
- Conduction mode, representing stable melt pool formation characterized by moderate penetration, adequate remelting of the deposited layer, and a balanced melt pool geometry. Stable conduction is identified when $0.30\leq D_{ref}/W_{ref}<0.65$, the predicted reference depth exceeds the deposited layer thickness, and the melt pool length-to-width ratio remains below 4 [29].
- Keyhole mode, corresponding to excessive laser penetration associated with deep and narrow melt pools. Keyhole behavior is identified when $D_{ref}/W_{ref}\geq 0.75$ together with a reference melt pool depth exceeding approximately 1.5 times the deposited layer thickness, consistent with the experimentally observed keyhole morphologies [30].
- Transition Region, representing intermediate conditions between stable conduction and keyhole melting. This regime captures melt pools exhibiting gradual morphological changes that do not satisfy the criteria for either stable conduction or fully developed keyhole behavior [31].
- Balling, identified when the melt pool length-to-width ratio exceeds a critical threshold of 4 [29,30,31].

Unlike conventional geometry-based classification methods that employ fixed empirical thresholds, the present criteria were refined using the experimentally observed melt pool morphologies obtained from the validated single-track experiments. This experimental calibration ensures that the identified processing regimes accurately reproduce the observed transitions within the investigated NiTi processing window while remaining physically interpretable.

The adopted dimensionless ratios represent engineering indicators of melt pool stability rather than universal material constants. The reference depth-to-width ratio describes the relative balance between penetration depth and lateral heat conduction, while the melt pool length-to-width ratio provides an indication of melt pool stability and thermal spreading. The selected thresholds therefore represent experimentally calibrated criteria specific to the investigated NiTi PBF-LB process and should be interpreted as process-dependent stability indicators rather than universally applicable limits.

By combining experimentally validated melt pool morphology with physics-informed decision rules, the digital twin extends beyond purely data-driven prediction by providing both quantitative estimates of melt pool geometry and qualitative assessment of processing stability. Consequently, the framework functions as an interpretable decision-support tool capable of identifying stable processing windows while maintaining direct physical correspondence with experimentally observed melt pool behavior.

### 3.6. Physics-Guided Decision Engine and Input Constraints

A key contribution of this work is the integration of physics-guided constraints within the decision-engine framework. Input parameters are restricted to discrete values corresponding to the experimentally and numerically validated DOE space used for surrogate model development, thereby preventing extrapolation beyond the surrogate’s reliable operating domain. This constraint-based strategy ensures that all predictions and parameter-search results remain physically meaningful and manufacturable.

By enforcing both numerical validity and discrete parameter constraints, the system operates as a controlled decision-support tool rather than an unconstrained predictive model. This design choice is particularly important for additive manufacturing applications, where small deviations in process parameters can lead to unstable melt pool behavior or defect formation [32].

### 3.7. Physics-Constrained Process Parameter Search

The developed framework enables automated process parameter exploration through a physics-constrained parameter-search routine aimed at identifying conduction-mode operating conditions. The decision engine leverages the surrogate model to rapidly evaluate candidate parameter combinations while rejecting solutions that violate melt pool stability criteria.

Unlike unconstrained parameter-selection approaches, the proposed decision engine prioritizes physical feasibility over purely mathematical optimality. As a result, optimized solutions consistently satisfy conduction-mode conditions while maintaining realistic laser power and scan speed combinations. This demonstrates the suitability of the developed system for use as a supervisory decision-support tool in additive manufacturing process planning.

### 3.8. Physics-Guided Process Maps and Experimental Validation

**Figure 11:**
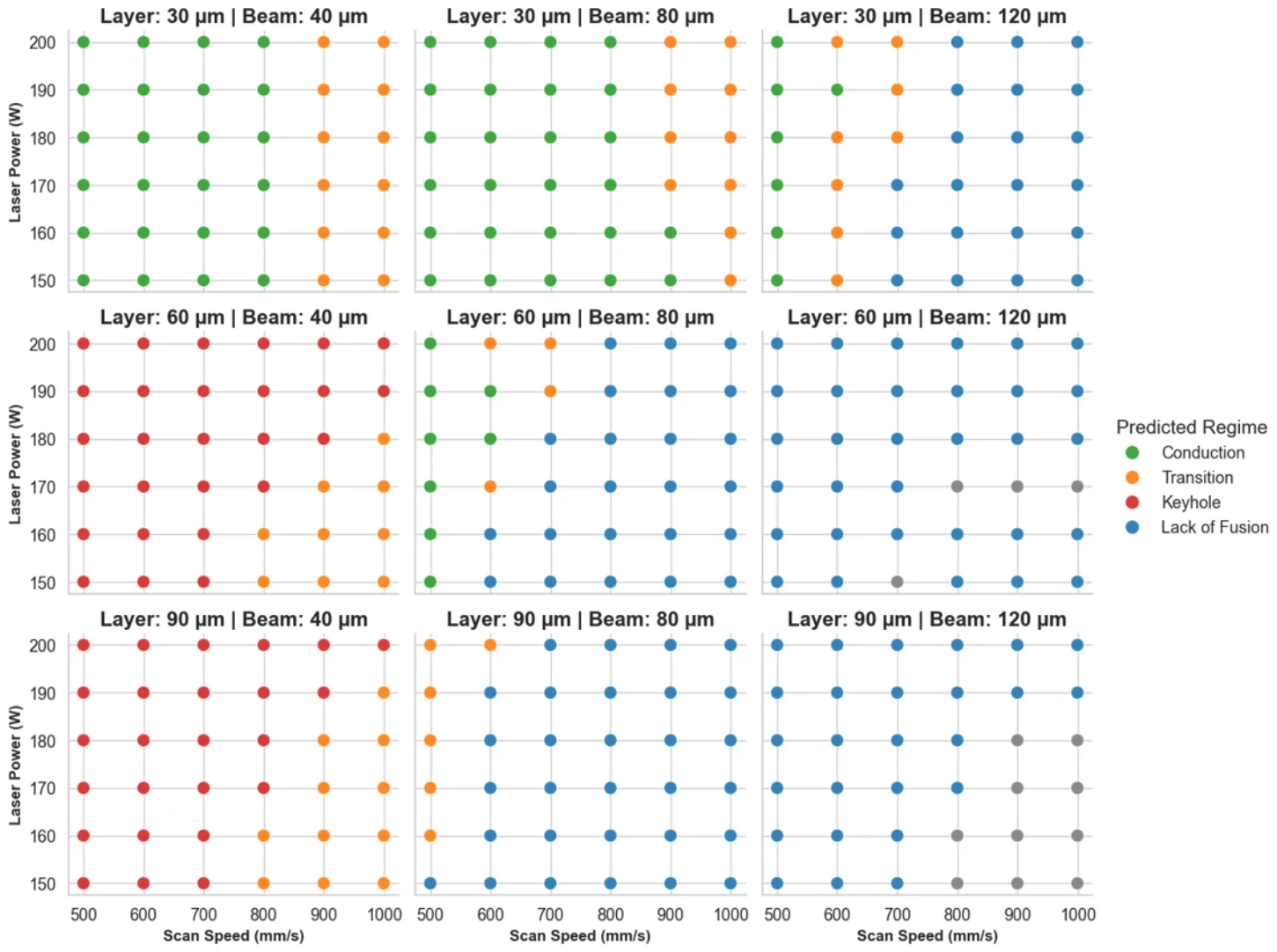
Physics-guided NiTi PBF-LB process maps predicted using the framework for different combinations of layer thickness and beam diameter. The process map corresponding to a layer thickness of 30 µm and beam diameter of 80 µm represents the experimentally investigated parameter space used for validation, while the remaining maps illustrate the framework predictions for extended processing conditions.

The physics-guided process maps generated using the proposed digital twin framework are presented in Figure 11. Each marker represents an individual process parameter combination evaluated by the proposed framework, with the corresponding melt pool regime determined using the experimentally calibrated, rule-based classification described in Section 3.5. The classification integrates predicted melt pool geometry with physics-based criteria derived from the experimentally observed melt pool morphologies, enabling each condition to be identified as conduction, transition, keyhole, or lack of fusion.

The process map corresponding to a 30 μm layer thickness and 80 μm beam diameter represents the experimentally validated parameter space and is based on the 36 single-track experiments performed over the laser power range of 150–200 W and scan speed range of 500–1000 mm/s. The experimental melt pool cross-sections were used to calibrate and verify the regime classification criteria, ensuring that the predicted processing regimes were consistent with the observed melt pool morphologies. Good agreement was observed between the experimentally identified melt pool characteristics and the corresponding predictions, demonstrating that the proposed physics-guided classification accurately captures transitions between stable and unstable melting behavior within the investigated NiTi processing window.

The calibrated classification framework was applied to generate process maps for additional combinations of layer thickness (30, 60, and 90 μm) and beam diameter (40, 80, and 120 μm). These extended maps were generated entirely using surrogate model predictions while employing the same experimentally validated classification criteria. Together, they illustrate the influence of processing parameters on melt pool stability beyond the experimentally investigated conditions and demonstrate the capability of the framework to rapidly explore a wider process design space without requiring additional physical experiments.

**Figure 12:**
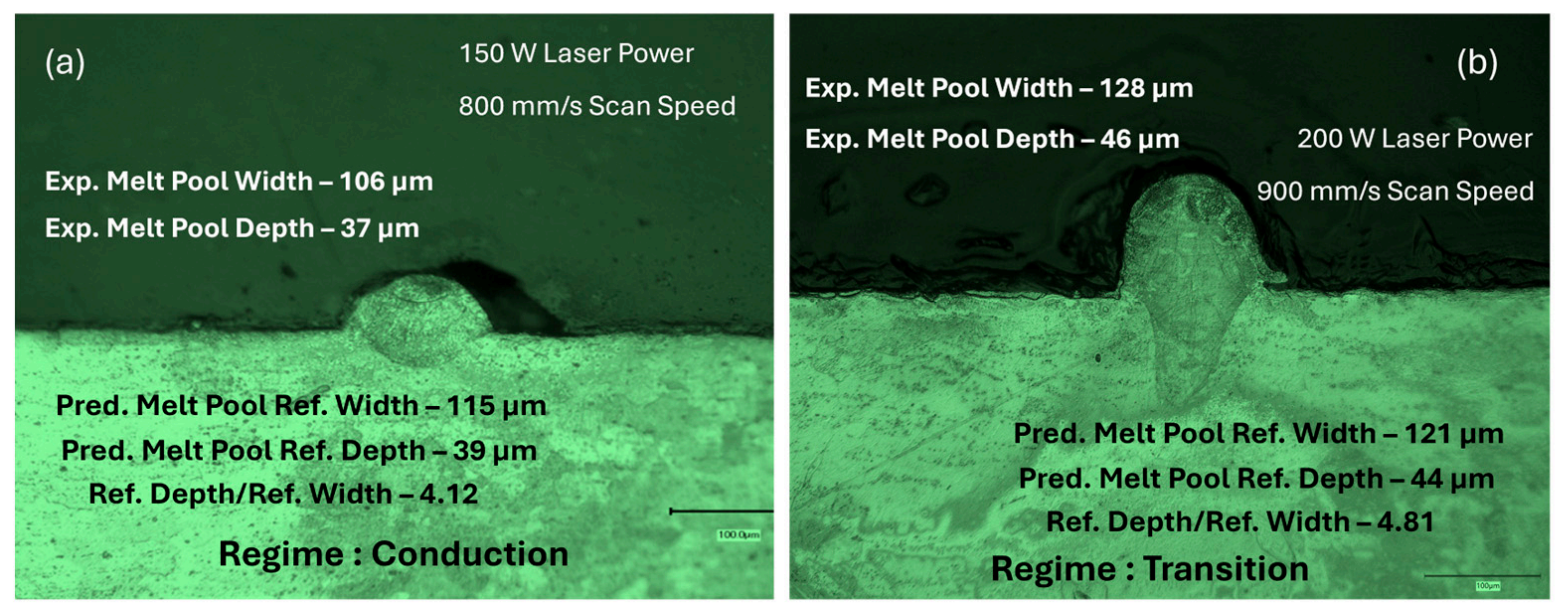
Representative experimentally observed melt pool cross-sections used to calibrate the physics-guided regime classification. The images illustrate the characteristic morphology associated with (**a**) stable conduction melting, and (**b**) transition melting. The corresponding processing regimes were identified using the experimentally calibrated geometric criteria adopted within the framework.

To demonstrate the physical basis of the proposed classification framework, representative experimentally observed melt pool morphologies corresponding to the principal processing regimes are presented in Figure 12. These micrographs were selected from the 36 single-track experiments used for model validation and illustrate the characteristic geometric features associated with stable conduction and transition regimes. The experimentally observed morphologies were used to calibrate the rule-based regime classification described in Section 3.5, thereby ensuring that the process maps reflect physically observed melt pool behavior rather than purely numerical predictions.

Overall, the proposed process mapping framework provides a significant advantage over conventional data-driven approaches by integrating experimentally validated melt pool morphology with physics-guided decision rules. Rather than merely interpolating between experimental observations, the framework generates interpretable process maps that identify stable processing windows, recommend optimal process parameters, and provide confidence for process selection across parameter combinations beyond those directly investigated experimentally. Consequently, the framework serves not only as a predictive model for melt pool geometry but also as a practical decision-support tool for process optimization and parameter selection in PBF-LB of NiTi.

### 3.9. Offline Digital Twin Interpretation and Implications

The integration of experimentally calibrated thermal modeling, surrogate modeling, physics-based regime classification, and constrained optimization establishes a physics-guided process-level decision-support framework for PBF-LB of NiTi. The present implementation operates offline and provides rapid predictive and decision-support capabilities within a validated process window. Although real-time sensing and adaptive model updating are not yet incorporated, the modular architecture has been designed to facilitate future integration with in situ monitoring and supervisory closed-loop control.

The present framework is based on a transient thermal FE model and therefore does not explicitly account for melt pool fluid flow, Marangoni convection, recoil pressure, or evaporation. These phenomena can influence melt pool geometry, particularly melt pool length, keyhole formation, and free-surface dynamics under high energy-density processing conditions [25]. Nevertheless, the thermal model was calibrated and independently validated using experimental melt pool measurements, enabling the dominant thermal behavior governing melt pool width and depth to be accurately captured within the investigated process window. Consequently, the proposed framework is well suited for rapid process prediction and parameter selection within the validated operating domain. Future developments will focus on incorporating thermo-fluid physics and in-situ monitoring to improve prediction accuracy under more extreme processing conditions and further extend the applicability of the developed system.

**Figure 13:**
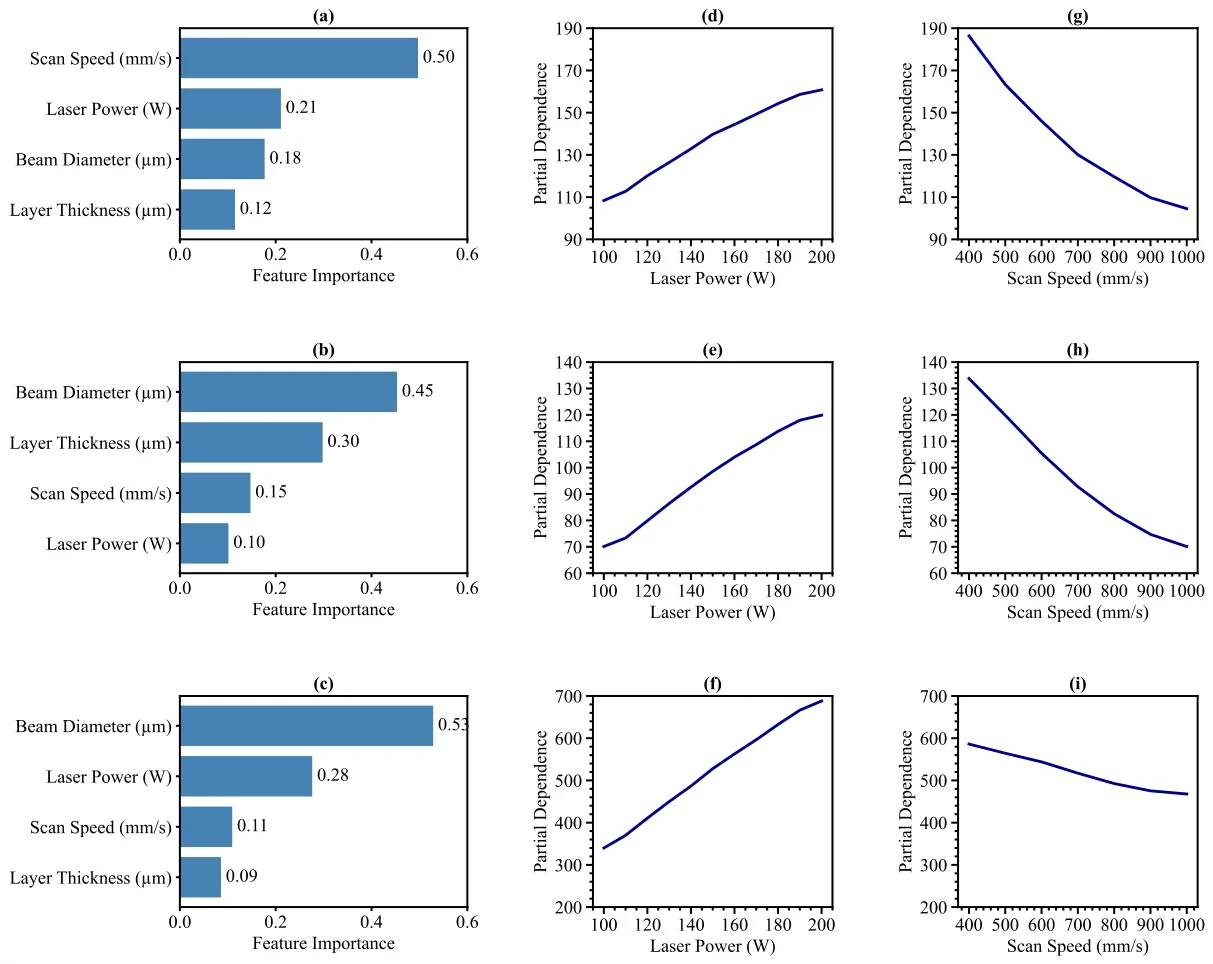
Random forest feature importance plots for melt pool (**a**) width, (**b**) depth, and (**c**) length and partial dependence plots showing the influence of laser power on melt pool (**d**) width, (**e**) depth, and (**f**) length, and of scan speed on melt pool (**g**) width, (**h**) depth, and (**i**) length.

Figure 13 shows the feature importance and partial dependence plots used to evaluate the effects of various PBF-LB process parameters on melt pool geometry. The feature importance analysis reveals that different melt pool dimensions are governed by distinct process parameters, highlighting the multi-physics nature of laser-matter interaction in PBF-LB [33]. Melt pool width is primarily controlled by scan speed and laser power, reflecting the role of energy input per unit length and lateral heat diffusion [31,34]. In contrast, melt pool depth is dominated by beam diameter and layer thickness, indicating the importance of power density and penetration efficiency [30,35]. Melt pool length is most sensitive to beam diameter and laser power, consistent with thermal trailing and solidification dynamics [32,36].

Partial dependence plots further demonstrate monotonic and physically consistent trends, with increasing laser power enhancing all melt pool dimensions, while increasing scan speed systematically reduces width, depth, and length as a result of decreased interaction time and energy density [37,38]. The trends corroborate well with the parameter-temperature relationship from the IR data and experimental analysis illustrated in Figure 5 and Figure 6, respectively. These trends demonstrate physically consistent surrogate behavior and provide a mechanistic basis for regime-based process optimization.

From a materials perspective, the predicted melt pool geometries are directly linked to solidification conditions, thermal gradients, and cooling rates, which govern microstructure evolution, defect formation, and phase transformation behavior in NiTi. In particular, the transition between conduction, keyhole, balling, and lack-of-fusion regimes provides a physically meaningful basis for identifying process windows that minimize defects and ensure stable processing conditions [24,39,40]. The incorporation of such physics-based criteria within the system enables not only prediction but also informed decision-making aligned with materials performance requirements.

Importantly, the framework is inherently extensible. While the present study focuses on offline prediction and optimization, the architecture is adaptable to closed-loop or supervisory control by incorporating in situ sensing and feedback. In this context, the surrogate model serves as the predictive core, while the physics-based decision logic ensures safe and stable operation.

Overall, the results demonstrate that integrating physics-guided regime criteria with data-driven surrogate modeling yields a robust, interpretable, and computationally efficient decision-support framework. The proposed approach enhances process understanding while providing a scalable foundation for future integration of relationships among process parameters, microstructure, and functional performance in NiTi additive manufacturing.

The offline digital twin was implemented as a modular Python framework consisting of dedicated prediction, regime-classification, and decision-engine components (see Supplementary Materials). This architecture enables reproducible deployment across graphical user interfaces, parameter-search routines, and future supervisory control extensions.

### 3.10. Limitations and Future Outlook

Several limitations of the present study should be acknowledged. First, the surrogate model is trained exclusively on simulation data and is therefore limited by the assumptions and fidelity of the underlying thermal model. Second, the current regime classification relies solely on geometric criteria and does not explicitly account for fluid flow, vaporization, or spatter dynamics, which may influence melt pool stability under extreme processing conditions [41,42].

It should also be noted that the experimental validation was conducted for a fixed beam diameter and layer thickness, whereas the extended process parameter space incorporated in the surrogate model was generated using the calibrated and independently validated FE thermal model. Consequently, predictions for additional beam diameters and layer thicknesses have been validated numerically but not yet verified through dedicated experiments. Furthermore, the surrogate model is intended for interpolation within this validated process window rather than extrapolation beyond it. Accordingly, the developed tool restricts user inputs to the predefined design-of-experiments levels of layer thickness, beam diameter, laser power, and scan speed. While random train–test splitting and five-fold cross-validation provide an appropriate assessment of interpolation accuracy within this bounded domain, experimental validation across the full parameter space and more stringent validation strategies, such as leave-one-level-out evaluation, remain important directions for future work.

The current framework represents an offline implementation of a physics-guided digital twin, in which process predictions are generated from an experimentally calibrated FE model and an associated machine-learning surrogate. Although the present study does not incorporate continuous synchronization with manufacturing data, real-time parameter updating, or autonomous feedback control, the modular architecture provides the computational foundation required for these capabilities.

Future work will therefore focus on integrating hybrid simulation–experimental datasets and in situ monitoring data to further improve physical representativeness. Furthermore, the framework can be extended to part-scale predictions, including porosity formation, surface roughness, and residual stress development. Additionally, integration with in situ sensing modalities such as melt pool imaging or pyrometry would enable closed-loop control and continuous model updating. These extensions would further enhance the applicability of the proposed framework for real-world additive manufacturing environments.

## 4. Conclusions

In this study, an offline physics-guided decision-support framework was developed for predicting melt pool behavior and supporting process parameter selection in PBF-LB of NiTi. A high-fidelity thermal finite element model was first established and calibrated using single-track experimental measurements and temperature-dependent thermophysical properties obtained via the CALPHAD method. The calibrated thermal model demonstrated strong agreement with experimental observations, achieving prediction accuracies exceeding 90% for melt pool width and depth.

Leveraging the validated thermal model, an extensive dataset comprising melt pool width, depth, and length was generated over a broad range of process parameters. This dataset was subsequently employed to train a multi-output random forest surrogate model capable of rapidly predicting melt pool geometric features with high computational efficiency. The surrogate demonstrated excellent predictive performance, achieving coefficients of determination (R^2^) greater than 0.95 for all melt pool descriptors, along with low mean absolute error (MAE) and root mean square error (RMSE) relative to the thermal model outputs.

To ensure physical interpretability and manufacturability, physics-based geometric criteria were embedded into the framework to classify melt pool regimes, including lack of fusion, keyhole mode, balling, and stable conduction. By integrating these criteria with surrogate predictions, the physics-guided system functions as a decision engine that identifies process parameter combinations conducive to stable conduction-mode melting. A graphical user interface was developed to facilitate intuitive interaction with the framework and to support informed process parameter selection.

While the current implementation is focused on micro-scale melt pool predictions, the proposed framework is inherently extensible. Future work will target expansion to meso-scale, micro-scale, and part-scale predictions, including porosity formation, surface roughness, residual stress development, microstructure, phase fractions, and resulting functional properties, thereby enhancing its relevance for component-level performance assessment. Furthermore, integration of the offline digital twin with in situ sensing and manufacturing hardware would enable closed-loop process control, paving the way toward adaptive and intelligent additive manufacturing systems. Additional experimental validation over the extended parameter space will further assess the robustness and generalizability of both the thermal and surrogate models.

Overall, this work demonstrates that coupling high-fidelity thermal modeling with data-driven surrogates and physics-based decision logic provides a powerful and scalable approach for melt pool prediction, regime identification, and process parameter selection in PBF-LB of NiTi.

## Figures and Tables

**Table 1:** Numerical simulation settings and calibrated heat-source parameters used in the Ansys AMPP thermal model.

Parameter	Value/Initial	Calibrated/Final
Software	Ansys AMPP 2025 R2	–
Mesh type	Structured Cartesian	–
X–Y mesh size	15 μm	–
Z mesh size	Automatically adjusted according to layer thickness	Comparable to X–Y mesh size
Time step	20 μs (2 × 10^−5^ s)	–
Initial powder/baseplate temperature	25 °C	–
Purge-gas convection coefficient	12.5 W m^−2^ K^−1^	12.5 W m^−2^ K^−1^
Solidus temperature	1580 °C	1580 °C
Liquidus temperature	1590 °C	1590 °C
Calibration dataset	Samples 10–18	–
Validation dataset	Samples 1–9 and 19–36	–
Absorptivity coefficient A	0.0000	−0.4523
Absorptivity coefficient B	0.0000	0.8970
Penetration depth coefficient A	0.0000	0.4268
Penetration depth coefficient B	0.0000	−1.1610

**Table 2:** Comparison of experimentally measured and FE thermal model-predicted melt pool geometries for the 36 single-track processing conditions. Samples 10–18 were used for thermal model calibration (in red), whereas Samples 1–9 and 19–36 were reserved for independent validation (in green). Percentage errors are reported relative to the experimental measurements.

Sample No.	Laser Power (W)	Scan Speed (mm/s)	Exp. Melt Pool Width (µm)	Model Melt Pool Ref Width (µm)	Width Error (%)	Exp. Melt Pool Depth (µm)	Model Melt Pool Ref Depth (µm)	Depth Error (%)	Melt Pool Width (µm)	Melt Pool Depth (µm)	Melt Pool Length (µm)
1	150	500	138	152	10.1	52	57	9.4	173	87	567
2	150	600	127	139	9.7	47	51	7.9	163	81	578
3	150	700	116	126	9.0	42	45	7.3	151	75	578
4	150	800	106	115	8.0	37	40	7.5	140	70	570
5	150	900	97	105	8.5	32	34	7.2	133	64	559
6	150	1000	88	95	7.5	28	29	4.7	125	59	544
7	160	500	143	156	9.1	53	59	11.1	178	89	588
8	160	600	130	143	9.9	48	53	9.4	166	83	601
9	160	700	120	131	8.9	43	47	10.3	156	77	603
10	160	800	109	119	9.3	39	42	7.5	142	72	598
11	160	900	100	110	10.0	35	37	6.0	137	67	593
12	160	1000	92	100	8.6	30	32	6.0	129	62	577
13	170	500	147	160	8.7	55	60	9.6	183	90	606
14	170	600	136	146	7.6	50	54	8.6	169	85	623
15	170	700	125	135	7.8	45	50	10.2	159	80	629
16	170	800	114	123	8.3	41	44	7.9	147	74	628
17	170	900	105	114	8.4	36	40	10.2	139	70	626
18	170	1000	96	105	8.9	32	34	7.6	132	64	610
19	180	500	161	163	1.2	56	61	9.4	186	91	622
20	180	600	150	150	−0.2	52	56	7.6	171	86	642
21	180	700	139	139	−0.3	47	51	8.6	163	81	654
22	180	800	128	128	−0.1	43	46	7.8	152	76	655
23	180	900	117	117	0.3	38	42	9.3	141	72	652
24	180	1000	106	109	2.9	34	37	8.7	136	67	644
25	190	500	168	166	−1.2	59	62	5.4	189	92	636
26	190	600	157	153	−2.8	55	57	4.5	173	88	661
27	190	700	145	141	−2.5	51	53	3.4	165	83	676
28	190	800	134	131	−1.9	47	48	2.8	157	78	681
29	190	900	123	121	−1.5	42	44	3.9	145	74	681
30	190	1000	112	112	0.4	38	39	3.5	138	69	675
31	200	500	174	169	−3.0	63	63	−0.1	191	93	650
32	200	600	163	156	−4.5	59	58	−0.9	177	89	678
33	200	700	150	144	−4.0	54	54	0.5	167	84	697
34	200	800	138	135	−2.2	50	50	0.1	159	80	706
35	200	900	128	125	−2.7	46	46	−0.9	149	76	708
36	200	1000	118	115	−2.1	42	41	−2.2	140	71	701

**Table 3:** Statistical performance metrics of the calibrated FE thermal model for the calibration and independent validation datasets. The reported MAE, RMSE, MBE, MAPE, and maximum percentage errors demonstrate the predictive accuracy of the model for melt pool width and depth.

Dataset	Width MAE (µm)	Width RMSE (µm)	Width MBE (µm)	Width MAPE (%)	Max Width Error (%)	Depth MAE (µm)	Depth RMSE (µm)	Depth MBE (µm)	Depth MAPE (%)	Max Depth Error (%)
Calibration (Runs 10–18)	9.78	9.87	+9.78	8.66	10.0	3.33	3.53	+3.33	8.11	10.2
Independent validation (Runs 1–9, 19–36)	5.33	6.92	+2.22	4.22	10.1	2.56	3.09	+2.41	5.63	11.1
Overall (All 36 runs)	6.44	7.76	+4.11	5.33	10.1	2.75	3.20	+2.64	6.25	11.1

## Data Availability

The original contributions presented in this study are included in the article/Supplementary Materials. Further inquiries can be directed to the corresponding authors.

## Supplementary Materials

The following supporting information can be downloaded at: https://github.com/sampreet-rangaswamy/Physics-Guided-Digital-Twin-LPBF-NiTi.git (accessed on 5 August 2026).

## Appendix A

**Table A1:** Summary of symbols, model inputs, outputs, and optimization variables used throughout the digital twin framework.

Symbol	Description	Unit
(h)	Layer thickness	µm
(d_b_)	Beam diameter	µm
(P)	Laser power	W
(v)	Scan speed	mm s^−1^
(W)	Melt pool width	µm
(D)	Melt pool depth	µm
Wref	Melt pool reference width	µm
Dref	Melt pool reference depth	µm
L	Melt pool length	µm
R_DW_	Reference depth-to-width ratio (Dref/Wref)	–
R_LW_	Length-to-width ratio ((L/W))	–
x	Input parameter vector	–
y^	Predicted melt pool geometry vector	–
f^(x)	Random Forest surrogate model	–
z	Optimization variable vector	–
J(z)	Stability objective function	–

**Table A2:** List of abbreviations used in the manuscript.

Abbreviation	Definition
AM	Additive Manufacturing
AMPP	Additive Manufacturing Process Parameters
CALPHAD	CALculation of PHAse Diagrams
DOE	Design of Experiments
FE	Finite Element
FEM	Finite Element Model
GUI	Graphical User Interface
LPBF	Laser Powder Bed Fusion
PBF-LB	Powder Bed Fusion–Laser Beam
MAE	Mean Absolute Error
RMSE	Root Mean Square Error
MAPE	Mean Absolute Percentage Error
MBE	Mean Bias Error
ML	Machine Learning
RF	Random Forest
(R^2^)	Coefficient of Determination
